# Stability Constant and Potentiometric Sensitivity of Heavy Metal–Organic Fluorescent Compound Complexes: QSPR Models for Prediction and Design of Novel Coumarin-like Ligands

**DOI:** 10.3390/toxics11070595

**Published:** 2023-07-07

**Authors:** Phan Thi Diem-Tran, Tue-Tam Ho, Nguyen-Van Tuan, Le-Quang Bao, Ha Tran Phuong, Trinh Thi Giao Chau, Hoang Thi Binh Minh, Cong-Truong Nguyen, Zulayho Smanova, Gerardo M. Casanola-Martin, Bakhtiyor Rasulev, Hai Pham-The, Le Canh Viet Cuong

**Affiliations:** 1 Mientrung Institute for Scientific Research, Vietnam National Museum of Nature, Vietnam Academy of Science and Technology, Hue 53000, Vietnam; 2 Faculty of Pharmaceutical Chemistry and Technology, Hanoi University of Pharmacy, 13-15 Le Thanh Tong, Hoan Kiem, Hanoi 10000, Vietnam; 3 Department of Chemistry, National University of Uzbekistan after Mirzo Ulugbek, Tashkent 100012, Uzbekistan; 4 Department of Coatings and Polymeric Materials, North Dakota State University, Fargo, ND 58102, USA

**Keywords:** florescent chemosensor, QSPR, quantum DFT calculation, stability constant, potentiometric sensitivity, toxicity, coumarin

## Abstract

Industrial wastewater often consists of toxic chemicals and pollutants, which are extremely harmful to the environment. Heavy metals are toxic chemicals and considered one of the major hazards to the aquatic ecosystem. Analytical techniques, such as potentiometric methods, are some of the methods to detect heavy metals in wastewaters. In this work, the quantitative structure–property relationship (QSPR) was applied using a range of machine learning techniques to predict the stability constant (logβ_ML_) and potentiometric sensitivity (PS_ML_) of 200 ligands in complexes with the heavy metal ions Cu^2+^, Cd^2+^, and Pb^2+^. In result, the logβML models developed for four ions showed good performance with square correlation coefficients (R^2^) ranging from 0.80 to 1.00 for the training and 0.72 to 0.85 for the test sets. Likewise, the PSML displayed acceptable performance with an R^2^ of 0.87 to 1.00 for the training and 0.73 to 0.95 for the test sets. By screening a virtual database of coumarin-like structures, several new ligands bearing the coumarin moiety were identified. Three of them, namely NEW02, NEW03, and NEW07, showed very good sensitivity and stability in the metal complexes. Subsequent quantum-chemical calculations, as well as physicochemical/toxicological profiling were performed to investigate their metal-binding ability and developability of the designed sensors. Finally, synthesis schemes are proposed to obtain these three ligands with major efficiency from simple resources. The three coumarins designed clearly demonstrated capability to be suitable as good florescent chemosensors towards heavy metals. Overall, the computational methods applied in this study showed a very good performance as useful tools for designing novel fluorescent probes and assessing their sensing abilities.

## 1. Introduction

The aggressive increase of industrial areas without proper waste management systems along rivers has created a huge source of water pollution [1,2,3]. Industrial wastewater normally consists of toxic chemicals and pollutants, which are extremely harmful to people and the environment [4]. Among all the pollutants from industry, heavy metals are considered as one of the major hazards to the aquatic ecosystem as they are poisonous and nonbiodegradable in nature, even at very low concentrations [5]. Elements that have high density and are less noxious are known as heavy metals. Generally, they are transition or basic metals with a density of >5 g/m^3^ [6]. They can easily migrate through the water–soil interface and the water–atmosphere interactions. There are various types of metal ions inside effluents including those of chromium (Cr(III) or Cr(IV)), manganese (Mn(II) or Mn(IV)), iron (Fe(II)), copper (Cu(II)), cadmium (Cd(II)), and lead (Pb(II)), among others, which can cause serious problems to the environment and living systems [5,7]. For example, long-term exposure to the ion Cd^2+^ with a higher concentration of 0.2 mg/kg through air, water, soil, and food leads to cancer and organ system toxicity such as the skeletal, urinary, reproductive, cardiovascular, central and peripheral nervous, and respiratory systems [8,9]. According to the World Health Organization (WHO), Pb^2+^ can attack the brain and central nervous system, causing coma, convulsions, and even death, at concentrations as low as 3.5 μg/dL [9]. Excessive intake of ion Cu^2+^ can cause liver or kidney damage [10]. A very recent report by Cao et al. showed a very alarming situation of water acidification and Cu^2+^ toxicity against estuarine ecosystems [11]. The above-mentioned examples clearly demonstrate an urgent demand for novel methods to detect and quantify heavy metals in the aqueous medium.

The analytical field has achieved significant advances over the last few decades in exploring the qualitative and quantitative information of pollutant elements [12,13]. Up to now, many techniques including ion-selective electrodes, reversed-phase high-performance liquid chromatography (HPLC), voltammetry, inductively coupled plasma mass spectroscopy (ICP-MS), plasma-atomic emission spectrometry (AES), and chemosensors (colorimetric, fluorometric, or fluorescent chemosensors) have been widely used in heavy metal ion detection [13]. Among them, fluorescent chemosensors have shown suitable detection specifications because of their visual simplicity, low cost, high sensitivity, good selectivity, and rapid response [14,15]. The first fluorescent chemosensor was introduced by Goppelsroder in 1867 for the determination of aluminum ions by forming a morin chelate with fluorescence-quenching effects. Since then, many fluorescent chemosensors have been developed for the detection of numerous different metal ions, which served as the precursors of analytical chemistry as widely known today [14]. In general, these sensors act as molecule receptors, which can sense and precisely interact with heavy metals and produce detectable signs due to the fluorescence intensity and/or fluorescence band shift. Designing such molecular systems for sensing metal ions through naked-eye detection is of high interest [15]. Different sensing mechanisms take place during sensing [12], such as fluorescence resonance energy transfer (FRET), intramolecular charge transfer (CT), chelation-enhanced fluorescence (CHEF), photoelectron transfer (PET), excimer formation, etc.

However, the conventional protocols for developing potential ligands able to selectively bind to the target ions require chemical synthesis and sensor membrane preparation and typically rely on the “trial and error” pathways by varying the compositions such as different solvent–plasticizers and different ratios between a ligand and an ion exchanger [16,17]. In addition, the sensitivity and selectivity parameters of the novel membranes must be examined by an appropriate potentiometric method. All these experiments require expensive, specialized, and cumbersome sample preparations and bulky laboratory equipment; thus, it is difficult to adapt them for multiple-metal-directed sensing applications or high-throughput screening of novel sensors [14,17,18,19].

In this sense, the quantitative structure–property relationship (QSPR) method [20,21,22,23,24,25,26,27,28], which is a computational approach composed by statistical models relating the structural features of ligands and their sensing properties, such as the stability constant or potentiometric sensitivity, have been employed to assist in the design and screening of fluorescence sensors [16,29,30,31,32]. Several achievements have been reported in this area, as summarized in Table 1. In 2019, Soloviev and colleagues developed several QSPR models able to predict the potentiometric sensitivity of sensors based on a variety of nitrogen-containing ligands (mainly diamides of pyridine and bipyridine acids) towards heavy metal ions (Cu^2+^, Zn^2+^, Cd^2+^, and Pb^2+^) with root-mean-squared errors of around 5 mV/dec based on the set of descriptors derived from substructural molecular fragments [29]. Later, Martynko et al. developed this concept further and aimed at Mg^2+^/Ca^2+^ potentiometric selectivity prediction using the QSPR based on the literature data [33]. While the attained precision of the model in prediction was not very high (±0.5 logKsel), the model was able to distinguish reliably between the ligands with high-, medium-, and low-Mg^2+^ concentration selectivity. Recently, Vladimirova and colleagues explored the potential of QSPR modeling in the prediction of potentiometric selectivity for plasticized polymeric membrane sensors based on newly synthesized ligands [30]. Attempts to develop novel QSPR models effectively applied for chemical sensors are ongoing and are needed to address the main issues currently posed in the field: (i) the limited number of data to be used for training together with the weak performance of the developed models and (ii) the lack of models to predict the multiple sensing properties of fluorescent probes and application for the virtual design of potential chemosensors.

In this work, for the first time, several QSPR models were developed in order to predict both the stability constant (β) and the potentiometric sensitivity (PS) of heterogeneous ionophores towards Cu^2+^, Cd^2+^, and Pb^2+^ ions simultaneously. The structure–property relationships (SPRs) extracted from the mathematical models were employed for designing novel coumarin fluorescent probes and assessing their sensing abilities. Finally, the newly designed ligands were evaluated for stability in complex by using quantum chemical calculations. The developability of the proposed chemosensors was also assessed by analyzing their toxicological profiles and effective organic synthesis routes.

## 2. Materials and Methods

### 2.1. Data Pre-Processing

The stability constant and potentiometric sensitivity database (Tables S1–S4 in the Supplementary Material) was compiled from numerous papers, which performed experiments to quantify these parameters for the three ions—cadmium, copper, and lead: Cd^2+^, Cu^2+^, and Pb^2+^. In fact, research manuscripts reporting large datasets that are deposited in a publicly available database should specify where the data were deposited and provide the relevant accession numbers. Importantly, only data obtained from the same experimental condition, such as temperature and pressure, were considered as having excluded external factors affecting the model. In this study, the data of two sensors’ properties were collected, including the stability constant (logβ) for Cd^2+^ [36,37,38,39], Cu^2+^ [40,41,42,43,44,45,46,47,48,49,50,51,52,53,54,55,56,57], and Pb^2+^ [40,58,59] and the potentiometric sensitivity (PS_ML_) [29,30].

The stability constant (β) shows how stable the complex formed between the metal and ligand is. The complex is more stable when this index rises [60]. The equation shown below is for the calculation of *β* when the ratio of the metal (*M*): ligand (*L*) is equal to 1:1.

(1) $$M+L\;\Leftrightarrow \;\;\;ML\;then\;\beta_{ML}=\frac{\left[ML\right]}{\left[M\right]\times \left[L\right]}$$

In this regard, *M* and *L* are also considered as Lewis acids and bases, and the complex stability could be explained using the hard/soft acids/bases (HSABs) principle by Pearson.

Potentiometric sensitivity (PS_ML_) is determined by the potential difference between reference and indicator electrodes, which are embedded in the individual aqueous solutions of metals in a range of concentrations [40]. It was calculated from the linear parts of the calibration curves obtained from the voltage values following each concentration of ion metal. The higher this index is, the larger the range of voltage potential exhibited. Defining this value will enable us to design more-accurate ion-selective membranes in the future. Each constant was also converted to the logarithm format for developing predictive models.

The ChemDraw 20.1.1 software was used to create the compound structures, which were then translated to the SMILES code so that the Dragon 6.0 software could calculate the molecular descriptors [61]. The data were preprocessed following the same protocols previously published [62,63]. Highly correlated descriptors (*R_ij_* > 0.95), constant and near constant (Std. < 0.1), were removed. The data were normalized to avoid bias while training the models. The standard scale normalization of the data was carried out using the Scikit-learn package based on the mean and standard deviation as follows [64]:

(2) $$x_{ij}=\frac{X_{ij}-{\bar{X}}_{j}}{\sqrt{\frac{\sum_{1}^{n}{\left(X_{ij}-{\bar{X}}_{j}\right)}^{2}}{n-1}}}$$

in which *n* is the number of compounds, *X_j_* is the mean values of the *j*th descriptor, and *x_ij_* and *X_ij_* are the normalized and original values of the *j*th descriptor of the *i*th compound. After cleaning the data, they were randomly divided into a training set and a test set for model construction or validation following the protocol implemented in the QSARINS Version 2.2.4 software [65].

### 2.2. Construction of QSPR Models Using Statistical and Machine Learning Techniques

The quantitative structure–properties relationship (QSPR) models in this study were built based on multi-linear regression (MLR) algorithms to predict the value of the logβ and PS_ML_ of the new compounds. In general, MLR can be represented as a linear combination of a subset of *X* variables (selected descriptor) that best describes the property *Y* with the regression coefficients αi and the intercept α_0_: $Y=\alpha_{0}+\alpha_{1}X_{1}+\ldots +\alpha_{n}X_{n}$. The optimization approach based on the genetic algorithm (GA) was used for descriptor selection and to calculate the linear parameters of the MLR models [66]. The GA-MLR is based on the principles of evolution in biology, which seeks optimal models by iteratively modifying a set of randomly generated variable combinations using genetic operators, i.e., crossover, mutation, etc. The *Q*^2^ under the influence of *K* (QUIK) rule in this algorithm enabled us to reduce the dimension of the model by setting a minimum threshold. If (*K_XY_* − *K_XX_*) *< δK*, this model will be excluded. *K_XY_* is the correlation between the independent variables and the dependent variable; *K_XX_* is total of correlation among the descriptors; *δK* is the threshold that the model trainer selects. This approach helped avoid multicollinearity. This entire procedure was carried out by using the QSARINS v2.2.4 software [65].

In the next steps, machine learning (ML) techniques were applied to examine the non-linear structure–property relationships (SPRs) of the chemical data. To do so, different ML algorithms implemented in the Scikit-learn library were employed using the same variable pools selected by the MLR models for each metal complex. Herein, ten algorithms, including non-linear regression (NR), the Gaussian process (GP), the decision tree (DT), support vector regression (SVR), multilayer perceptron (MLP), the *k* neighbors regressor (KNR), random forest (FR), the AdaBoost regressor MLR, the AdaBoost regressor DT, and the gradient boosting regressor were used to obtain ML models and then compared to the performance of the GA-MLR model [64]. At this stage, only the best model showing the highest performance in both the cross-validation and external validation was chosen for further screening assays. The runtime parameters corresponding to the ML models developed for each property are provided in Tables S5–S10 (Supplementary Materials). The correlation coefficient (*R*^2^) was calculated from the experimental values on the ordinate axis, and models with *R*^2^ < 0.6 were not included in this analysis [67].

#### 2.2.1. Performance Assessment

QSPR models were evaluated through some statistical parameters. The first one is *R*^2^, which determines the proportion of variance in the dependent variable that can be explained by the independent variable [68] and is calculated by this equation:

(3) $$R^{2}=1-\frac{\sum {\left(y_{i}-{\hat{y}}_{i}\right)}^{2}}{\sum {\left(y_{i}-{\bar{y}}_{i}\right)}^{2}}=\frac{\sum {\left({\hat{y}}_{i}-{\bar{y}}_{i}\right)}^{2}}{\sum {\left(y_{i}-{\bar{y}}_{i}\right)}^{2}}$$

*y*_i_ and ${\hat{y}}_{i}$ are the experimental and predictive values of the dependent variable, respectively; ${\bar{y}}_{i}$ is the average value of *y*_i_ (all of them are in the training set).

However, it should be noted that the MLR model’s *R*^2^ increased as the number of variables increased, which gradually resulted in overfitting. Therefore, the quality of the model was assessed using this adjusted parameter [68]:

(4) $$R_{adj}^{2}=1-(1-R^{2})\times (\frac{n-1}{n-p-1})$$

where the *n* and *p* parameters are the number of compounds and independent variables in the training set, respectively.

In addition, we also checked for the fitness of the MLR models during the evolution process by calculating the Friedman lack-of-fit (LOF) measurement [69]. According to the analysis of Bondarchuk [70,71], this parameter is more resistant to overfitting in comparison to the commonly used least-squares error.

(5) $$LOF=\frac{SSE}{N{\left[1-\lambda \left(\frac{c+dp}{N}\right)\right]}^{2}}$$

In this form, the *SSE* is the sum of squares of errors; *c* is the number of terms in the model; p is the total number of descriptors; *N* is the number of data rows in the training set; 𝜆 is a safety factor; *d* is a scaled smoothing parameter [70]. Accordingly, a model with a lower LOF value is better. However, there is no universally accepted threshold to determine what constitutes a low LOF value, as this can depend on many factors such as the nature of the data, the quantity of data, and the specific algorithm used. Therefore, to determine whether this value is acceptable or not, we performed the F-test of the LOF, taking into consideration the *p*-values (<0.01) as the F-statistic for the LOF. In this sense, a *p*-value < 0.01 indicates that the LOF of selected model is significantly lower than the other models, suggesting a better-fitting model.

#### 2.2.2. Internal Validation

*R*^2^ and $R_{adj}^{2}$ merely demonstrate the goodness-of-fit of QSPR models. Cross-validation strategies such as leave-one-out (LOO) and leave-many-out (LMO) were applied to ensure the model’s stability [72]. The result from the LOO approach is the cross-validation correlation coefficient $Q_{LOO}^{2}$, calculated by this formula:

(6) $$Q_{LOO}^{2}=1-\frac{\sum {\left(y_{i}-{\hat{y}}_{i/i}\right)}^{2}}{\sum {\left(y_{i}-{\bar{y}}_{i}\right)}^{2}}$$

*y*_i_ is the experimental property of the *i*th compound, and ${\hat{y}}_{i/i}$ is the predictive one from the model built by *n* − 1 remaining compounds (not including compound *i*).

LMO was also performed to overcome the disadvantages of the LOO method, which might exaggerate the predictive power of the model [73]. Then, 70% of the training set was randomly selected to construct the model for the prediction of the remaining 30%. This process was repeated 2000 times and the *Q*^2^ value calculated each time. These values should be close to $Q_{LOO}^{2}$ and to their mean ($Q_{LMO}^{2}$).

Another issue might come out that the model accidentally has the accuracy of prediction. To exclude this possibility, *Y-*randomization was applied by randomly rearranging the values of the dependent variables. Calculate *R*^2^*Y*, *Q*^2^*Y* each time 2000 times. These values should be far from the original value of the models.

#### 2.2.3. External Validation

The test set was used to validate the predictive ability of the model by calculating the indexes below [65,73]:

(7) $$Q_{F1}^{2}=1-\frac{\sum {\left(y_{i}-{\hat{y}}_{i}\right)}^{2}}{\sum {\left(y_{i}-{\bar{y}}_{Tr}\right)}^{2}}$$

(8) $$Q_{F2}^{2}=1-\frac{\sum {\left(y_{i}-{\hat{y}}_{i}\right)}^{2}}{\sum {\left(y_{i}-{\bar{y}}_{EXT}\right)}^{2}}$$

(9) $$Q_{F3}^{2}=1-\frac{\sum {\left(y_{i}-{\hat{y}}_{i}\right)}^{2}/n_{EXT}}{\sum {\left(y_{i}-{\bar{y}}_{Tr}\right)}^{2}/n_{Tr}}$$

where *y_i_* and ${\hat{y}}_{i/i}$ are the experimental and predictive values of the property of the compound *i*^th^, respectively; ${\bar{y}}_{Ext}$ is average of the experimental values of the property in the training set; $n_{Ext}$ and $n_{Tr}$ are the number of compounds in the test set and training set, respectively.

In general, if *R*^2^ > 0.6 and *Q*^2^ > 0.5, the model is considered to be a good one. The closer *R*^2^ and *Q*^2^ are to 1, the better the obtained models are.

### 2.3. Applicability Domain

The QSAR/QSPR models need to determine the applicability domain (AD). AD is an area defined by the molecular descriptors and response variables of the training set. We calculated the leverage value to determine if a substance belonged to the AD following this equation [74]:

(10) $$h_{i}=x_{i}^{T}{\left(X^{T}X\right)}^{-1}x_{i}$$

*h_i_* is the leverage value of the *i*th compound; *x*_i_ is the molecular descriptor of the compound; *X* is matrix including *k* variables (*k* columns) of *n* compounds (*n* rows).

The AD is limited by a line of value h* = 3*p*’/*n* with *p*’ as the number of variables of the model plus 1 and *n* as the number of compounds in the training set. If a compound has *h_i_* > h* and the predicted value is outside of three-times the standard residual, it may not be predicted well by the model, or the predictive value is not reliable in this circumstance [63].

### 2.4. Design of Novel Coumarin-like Structures

Coumarin fluorescent probes have received significant interest in recent years due to their favorable fluorescence properties, including a high fluorescence quantum yield, long emission wavelengths, and photostability [75]. In this study, several coumarin derivatives were designed based on the precursor probes previously reported using the Marvin Sketch R-group attachment module, ChemAxon (http://www.chemaxon.com, accessed on 5 November 2022). These scaffolds showed very good selectivity in the recognition of most competing metal ions, including Cd^2+^, Cu^2+^, and Pb^2+^. By modifying the groups that have a different effect on the efficiency of charge transfer and the electron configuration of the benzene and naphthalene rings of the coumarin derivatives, it is expected that compounds will be identified with the enhancement of the fluorescence intensity towards all three metals.

### 2.5. Quantum Chemical Calculations

Density functional theory (DFT) calculations were performed for the ground-state gas-phase geometry optimization of the metal complexes using Beck’s three-parameter exchange functional Lee–Yang–Parr (B3LYP) methods combined with the LanL2DZ basis set in the gas phase [76]. The consensus measurements were considered for estimating the stability of the complexes formed. The calculations of the newly designed compounds were performed by using the GAMESS quantum chemistry package, and the wide range of parameters were analyzed including the highest occupied molecular orbital (HOMO) and the lowest unoccupied molecular orbital (LUMO) energies, among others [77]. Besides computing the HOMO–LUMO gap ΔE = E_LUMO_ − E_HOMO_, the Mulliken electronegativity *χ* and hardness *η* values could be extracted based on the finite difference approximation, viz*. χ* = (IE + EA)/2 and *η* = (IE − EA)/2, where IE is the ionization energy or ionization potential and EA is the electron affinity of the ionic states of the complex system. Based on the semiquantitative approach proposed by Zhan et al., IE, EA, *χ*, and *η* can be estimated based on the calculated HOMO and LUMO energies [78]. Other properties of interest included the global softness *σ* = 1/*η*, which measures the extent of chemical reactivity, and the chemical potential of the complex system (*μ*), which is the first derivative of the total energy with respect to the number of electrons [79]. Parr and colleagues proposed a new global chemical reactivity parameter, namely an electrophilicity index (*ω*), which may be calculated via the equation *ω* = *μ*^2^/2*η* [80]. In order to take into account the diffuse characteristics of the molecular orbitals of heavy elements with a large electron density, all of these derived parameters were made at the B3LYP/LANL2DZ level.

### 2.6. Physicochemical and Toxicological Profiling

Given the broad application of the designed coumarin fluorescent probes, especially in an aqueous medium, it is of pivotal importance to determine the possible effects of the chemosensors for human and aquatic organism health. Important physicochemical properties mainly governing the aqueous solubility, organism permeability, and in vivo dermal and oral bioavailability were calculated using consensus cheminformatics approaches such as SwissADME [81] and ADMETlab 2.0 [82], among others [83,84,85,86]. To do so, chemical SMILES codes were used as the input of the computational tools, and the results were extracted based on more than 100 indices calculated previously. In addition, multiple toxicological endpoints of the newly developed sensors towards aquatic organisms were also investigated using the same tools [81,82].

### 2.7. Theoretical Proposal of Synthetic Accessibility for Promising Candidates

The prediction of the synthetic accessibility (SA) of the potential probe candidates was carried out using the GASA [87], ADMETlab 2.0 [82], and SwissADME tools [81]. GASA employs a rapid graph-based molecular synthetic accessibility prediction method, which has been demonstrated to be comparable to the existing methods, such as SYBA, SCScore, RAscore, and SAscore, among others. Based on the probability of the predictions, compounds can be classified as easy- (ES) or hard-to-synthesize (HS) structures [87]. This model was trained on 640,000 compounds and achieved an accuracy of 0.932 with an AUC of 0.984. In ADMETlab 2.0, the SA scores can be predicted with ~90% accuracy using the fragment-based approach developed by Ertl and Schuffenhauer [88], on the basis of 934,046 representative molecules from the PubChem database. In addition, the SwissADME tool allows calculating the SA scores based on a fragmental contribution approach. This method consists of the analysis of nearly 13 million molecules with the hypothesis that the more frequent a chemical fragment, the easier it is to synthesize that molecule [81]. The SA scores computed by SwissADME receive a value ranging from 1 (very easy synthesis) to 10 (very difficult synthesis).

## 3. Results and Discussion

### 3.1. Development and Selection of GA-MLR QSPR Models

A total number of six MLR models were constructed based on the experimental logβ and PS data. Applying an extensive feature selection process of >105 iterations by the GA, 90% of the new generations showed the same fitness results. The best MLR models were selected from the final iteration taking into account Austin’s criteria (5 × number of variables ≤ number of observations) to avoid the overfitting problem [66,89]. The best models were further analyzed and compared based on the quality of the statistical parameters, as shown in Table 2. The mathematical formulas of the six models are given by Equations (10) to (15), and more details about the variables selected can be found in Table S11, Supplementary Material.

(11) $$\begin{array}{l} Log\beta_{CdL}=9.2894-2.6191\times RBN+6.0044\times SPI+15.7303\times H\_D/Dt\\ \;\;\;\;-4.6761\times MATS1m-11.3909\times MATS2m+4.987\times MATS6i \end{array}$$

(12) $$\begin{array}{l} Log\beta_{CuL}=8.2894+7.1518\times nHM+5.5297\times ATSC4e+5.6081\times MATS1v\\ \;\;\;\;\;+9.7064\times MATS6p-10.5641\times MATS7p+7.4692\times GATS5p\;\\ \;\;\;\;\;+12.2912\times P\_VSA\_MR\_7-19.6671\times SM07\_AEA\left(ed\right)\\ \;\;\;\;\;+8.5756\times O-057+12.5774\times NssCH2-1.8575\times B06\left[C-O\right]? \end{array}$$

(13) $$\begin{array}{l} Log\beta_{PbL}=4.6561-1.1628\times nR05-6.1855\times J\_Dt+6.4076\times ATSC1s\\ \;\;\;\;\;+6.4211\times GATS8m-6.0816\times SM03\_EA\left(bo\right)\;\; \end{array}$$

(14) $$\begin{array}{l} PS_{CdL}=-32.5684-13.696\times J\_B\left(s\right)+16.1241\times GATS8i+27.1608\times JGI8\\ \;\;\;\;+30.3906\times SpMax4\_Bh\left(m\right)-21.3087\times SpMin3\_Bh\left(s\right)\\ \;\;\;\;+38.7644\times SpMin5\_Bh\left(s\right)+9.2121\times DLS\_01? \end{array}$$

(15) $$\begin{array}{l} PS_{CuL}=35.4214-16.7676\times Ho\_Dz\left(p\right)-12.1368\times GGI10\\ \;\;\;\;-29.2904\times P\_VSA\_p\_2+23.0366\times Chi1\_EA\left(dm\right)\\ \;\;\;\;+17.6022\times DLS\_04-42.4502\times LLS\_01? \end{array}$$

(16) $$\begin{array}{l} PS_{PbL}=-53.7619+16.311\times MATS6v+30.6395\times MATS2i\\ \;\;\;\;-10.6447\times GATS4i-50.3016\times GGI8+45.0039\times JGI8\\ \;\;\;\;+71.9904\times SpMin5\_Bh\left(s\right)+24.299\times Eta\_F\_A? \end{array}$$

**Figure 1:**
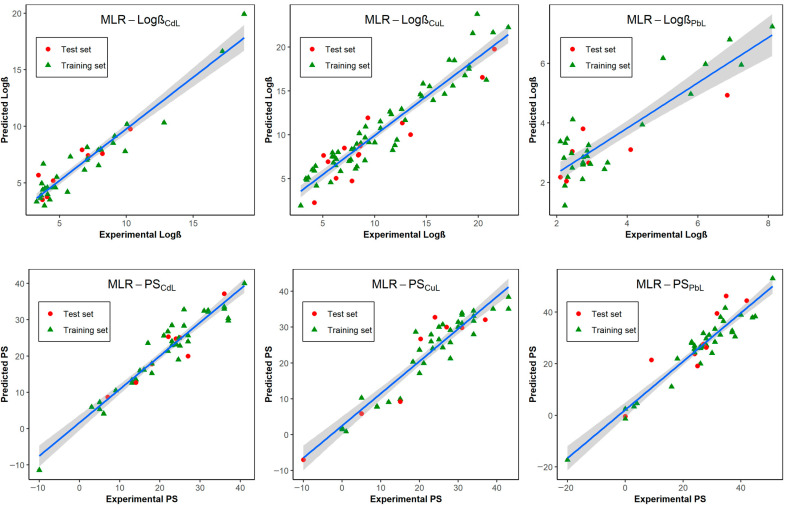
Scatter plots illustrating the correlation between the experimental and predictive values of the six QSPR models.

In general, all six models presented a goodness-of-fit with an *R*^2^ and $R_{adj}^{2}$ of approximately 0.9, except for the *logβ_PbL_*-only models, whose values were ~0.8 (Figure 1). According to the Friedman LOF parameters, the *logβ* model of the Cu complex showed the highest value of 7.05, and the lowest value corresponded to the *logβ* model of *Pb* (1.35). As can be seen in Table 2, the F-statistics for the F-test of the LOF were highly significant (*p*-value ranging from 1.095 × 10^−19^ to 3.087 × 10^−7^), suggesting an acceptable level of the LOF. From this test, we can confirm that no overfitting existed in the models, as they presented a good fit with a minimum number of descriptors. Considering the internal validation, the values of $Q_{LOO}^{2}$ for almost all models were greater than 0.8 with the model MLR-logβ_PbL_ being the lone exception, owning an approximate *Q*^2^ value of 0.7. For the LMO approach, the $Q_{LMO}^{2}$ values after each iteration showed a slight change, being around the $Q_{LOO}^{2}$ of the primary model (Mod. *Q*^2^) (see Figure S1). This indicated that the $Q_{LOO}^{2}$ index in these models does not overate their predictive ability. In addition to the above criteria, the optimal model was chosen based on the lower level of *R*^2^ −$Q_{LOO}^{2}$, the correlation among the independent variables (as low as possible), and the high correlation between the dependent variables and independent variable (high *δK* values). Alternatively, *Y* randomization also demonstrated that the accuracy of these models was not an accidental correlation. We found that *R*^2^, *Q*^2^, and *K_XY_* in each iteration were less than the original model (Figure S2). The autocorrelation between the descriptors was checked through the Pearson correlation matrix and the correlation among the descriptors (*K_XX_*). As the results show in Figures S3–S8, no significant correlation between the variables was detected, as the average values of the pairwise correlation ranged from 0.25–0.48. All the descriptor values calculated by the Dragon software for each of the training and test set compounds included in the six databases are provided in Tables S21–S26.

In the external validation, the $Q_{F1}^{2}$, $Q_{F2}^{2}$, and $Q_{F3}^{2}$ of the six models were almost higher than 0.8, except the Pb^2+^ models with $Q_{F1}^{2}$ values between 0.6 and 0.7, which are still acceptable according to our criteria (Table 2). From the results, all the MLR models were able to predict the logβ and PS_ML_ values of the unknown structures with high accuracy.

**Figure 2:**
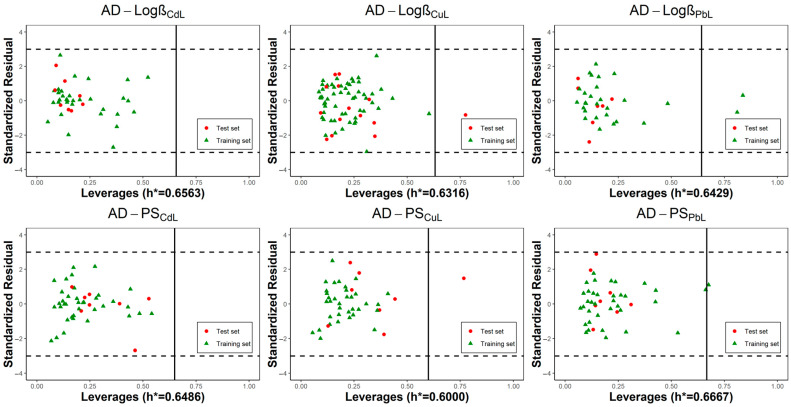
William plot showing the applicability domain of each model.

The applicability domain of each model is shown in Figure 2. If a new chemical is to be predicted, it must fall within the AD of the model. The outliers of the domain included two types: *Y* outlier and *X* outlier. The *Y* outlier indicates that the predictive value is not unreliable, and the *X* outlier shows that these compounds should not be applied to the model. There were no *Y* outliers in all six plots, so the quality of these models was ensured. Regarding the *X* outlier, there were a few compounds that are outside of the domain. Therefore, we need to pay more attention to these structures [63].

### 3.2. Machine Learning Algorithms for Improving Model Performance

**Figure 3:**
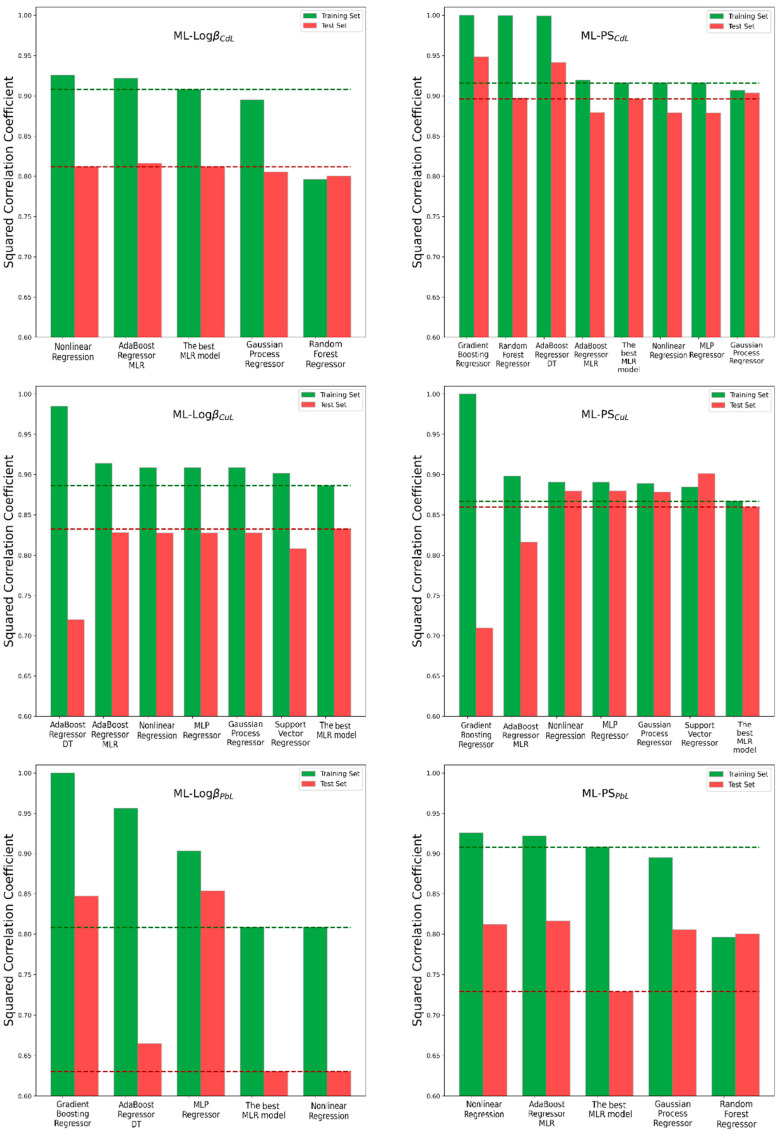
The accuracy comparison of the best MLR models and machine learning models. The green and red dashed lines indicate the accuracy from the training and external testing processes of the MLR models, respectively.

As can be revealed from the validation of the MLR models, the statistical linear algorithm still showed weak performances; for example, the models to predict the sensing properties of the Pb^2+^ complexes only had a testing *Q*^2^ of 0.64–0.71 (Table 2). To overcome this challenge, machine learning (ML) methods were applied using the same variable subset selected by the GA-MLR. A wide range of 10 ML algorithms were explored adopting the Scikit-learn package in Python using the same script we developed previously [64]. To optimize the model performance, a parameter tuning search was applied, and the entire results are listed in Tables S5–S10, the Supplementary Materials. Note that only models showing an accuracy higher than 0.6 were retained for this analysis, and the models with the highest performance were used for further virtual screening assays. Figure 3 displays the comparison histogram of every ML model against the best MLR models obtained previously. In general, there was a large dissimilarity among the modeling techniques, and the ML algorithms normally outperformed the simple linear models in both *k*-fold cross-validation on the training set and the external test set. A detailed comparison of all the selected regressors is shown in Tables S12–S17, and the resulting runtime parameters of the selected models are given in Table 3.

Regarding the models to predict the stability constant (logβ) values of the Cd^2+^–ligand complexes, the histogram shown in Figure 3 indicated that the accuracy of the AdaBoost regressor MLR and non-linear regression were comparable and higher than other models, being 0.922/0.820 and 0.926/0.812 for the training/testing *R*^2^, respectively. These results were slightly higher than the best MLR model, especially the training process, which resulted in an *R*^2^ value of 0.908. AdaBoost has been described as an ensemble boosting algorithm, which repeatedly combines regressors, i.e., MLR or decision trees (DTs), on the same training set with a special focus on the error of the current prediction and the difficult cases. Meanwhile, the non-linear regression algorithm, unlike the statistical linear estimator, allows for a more complex relationship between the dependent and independent variables [90]. Herein, three functions were tested, including the polynomial, logarithmic, and exponential regressions. Given the importance of external testing, the AdaBoost regressor MLR was selected for further use in the new case prediction.

For the potentiometric sensitivity (PS_ML_) data of the Cd^2+^–ligand complexes, seven ML models are shown, the gradient boosting, random forest, and AdaBoost DT being the regressors with the highest accuracy for the training set (*R*^2^ ~1.0). However, only gradient boosting and the AdaBoost DT significantly outperformed the other models with a testing *R*^2^ of 0.948 and 0.941, respectively. Unlike AdaBoost, gradient boosting uses the gradient decent algorithm to add the weak learners in a stagewise manner so that the overall prediction error significantly decreases. Consequently, the gradient boosting model was selected for the PS_ML_ value prediction of the Cd^2+^–ligand complexes.

In contrast to the Cd^2+^ complex data, most of the ML techniques showed good performance on the Cu^2+^ logβ and PS_ML_ data. For the logβ data, the AdaBoost regression DT significantly outperformed the other models on the training (*R*^2^ = 0.984), but only obtained an accuracy of 0.721 on the test set, which was much lower than the MLR model. In this case, MLR, even having a slightly lower coefficient for the training set (*R*^2^ = 0.89) in comparison with the other ML models (training *R*^2^ = 0.90–0.91), surprisingly exhibited the highest accuracy on the test set. We then selected the linear model MLR for further prediction of the Cu^2+^ logβ data.

The results of the ML models in predicting the PS data of the Cu^2+^–ligand complexes were somewhat the same situation as the Cu^2+^ logβ data. However, we identified support vector regression (SVR) as the best model, owning an accuracy of 0.884 and 0.901 for the training and test sets. In this case, MLR was the model with the lowest performance on both the training (training *R*^2^ = 0.867) and test sets (average testing *R*^2^ = 0.86). More details on the SVR algorithm implemented in the Scikit-learn package can be found in the literature [64]. This model was subsequently used in the next screening assays based on the Cu^2+^ PS_ML_ data.

The accuracy results from the Pb^2+^ logβ and PS_ML_ data were very encouraging. We found significant improvement when using ML techniques in comparison with the MLR model. For the Pb^2+^ logβ data, the gradient boosting regressor once again showed the highest performance, owing to the determinant coefficient for the training and test sets of 1.0 and 0.847, respectively. Another ML algorithm that also performed well on these data was multi-layer perceptron (MLP) with a training and testing accuracy of 0.903 and 0.853, respectively; however, the training results were lower than gradient boosting. Consequently, the gradient boosting model was without a doubt selected for further prediction of the Pb^2+^ logβ data.

For the Pb^2+^ PS_ML_ data, the AdaBoost regressor MLR displayed high performance with the accuracy on the training and testing sets being 0.922 and 0.816, respectively. One these data, the MLR model also performed well on the training set with an *R*^2^ value of 0.908, but the accuracy on the test set significantly dropped to ~0.7, resulting in the worst model in the external validation. Thus, the AdaBoost regressor MLR was selected for predicting the PS_ML_ values of the Pb^2+^–new ligand complexes.

### 3.3. Design of Coumarin-like Chemical Library for Screening of Novel Chemosensors

One of the main applications of QSPR/QSAR approaches is to screen novel active ligands from a large chemical library of a determined scaffold. Bearing in mind that coumarin fluorescence probes for heavy metal ions currently receive increasing interest due to their highly variable size, hydrophobicity, and chelation, in this study, we focused on the design and screening of novel fluorescent probes bearing coumarin moieties [75,91]. In addition, Schiff bases are attracting more and more attention in analytical fields as they provide an electron-rich environment that is favorable to binding with metal ions [92]. When combined with a fluorescence moiety such as coumarin structure, the functional groups can participate in several reactions after being in contact with metal ions so that the Schiff base ligand provides detectable signals such as emission.

In 2017, Fan reported the synthesis of several coumarin-derived Schiff bases, which showed significant fluorescence enhancement owing to inhibiting the photo-induced electron transfer (PET) process [93]. The structures were mainly based on condensing 7-amino-4-methyl-coumarin (AMC) with salicylic aldehyde and 2-hydroxy-1-naphthaldehyde. Fan also evaluated the selectivity of his derivatives upon the addition of several metal ions such as Cd^2+^ and Pb^2+^, among others (15 metals). The fluorescent property of these Schiff bases appeared to be dependent on the charge transfer and the electron configuration of the benzene and naphthalene rings. However, Cu^2+^ was not tested for the coumarins designed in Fan’s study [93]; thus, this core structure was explored in this study.

Going through the literature, we encountered coumarin-pyridin hybrids reported by Jung et al., which showed promising fluorescence-quenching properties in both the femtosecond time-resolved fluorescence (TRF) upconversion technique and ab initio calculations [47]. The authors identified that the n-picolylamide moiety (n = 1–3) allowed efficient tridentate complexation for Cu^2+^ preferred over a variety of other common heavy and toxic metal ions. Of interest is also the chemosensor bearing acetyl-7-hydroxycoumarin moiety reported by Chang and colleagues, which exhibited a highly selective and sensitive fluorescence-sensing ability for Cu^-+^ over other metal ions under physiological conditions [94]. We also consulted the most recent advances in the field from literature reviews [75,91].

**Figure 4:**
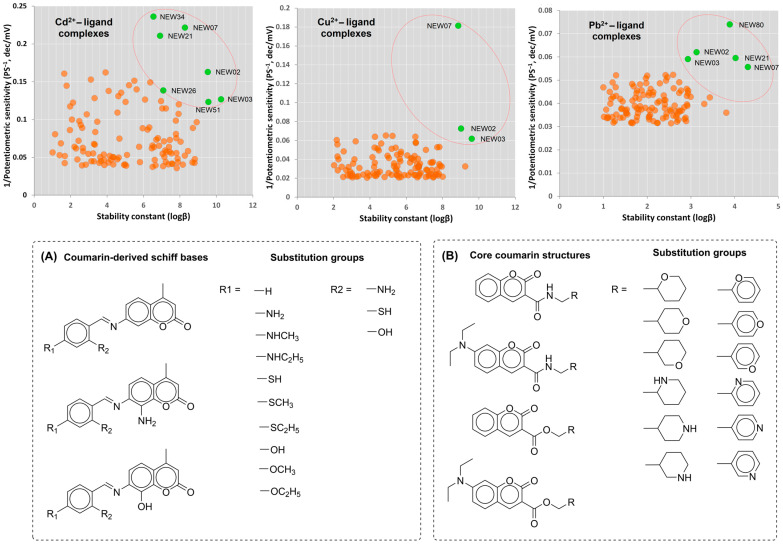
Design and screening of the virtual chemical library of coumarin derivatives as possible fluorescent probes. Chemical design was based on: (**A**) coumarin-derived Schiff bases, and (**B**) other core coumarin structures. Coumarins predicted as potential florescent probes are highlighted (green dots inside the circles) in comparison with the other compounds (orange dots).

Taking all the above-mentioned findings, here, we tried to construct a small library of coumarin derivatives by using a structure-resembling approach using the R-group attachment module implemented int the Marvin Sketch package. As a result, over 129 coumarin structures were created, as shown in Figure 4A,B. All newly designed substances were checked to see if they lied in the range of the AD. Their calculated leverage values (${\hat{h}}_{i}$) were all smaller than the threshold value (h*) of all the models, which means that QSPR models could work correctly on the chemical space of the designed compounds as they fell into the AD of the models.

### 3.4. Virtual Screening and Complexation Potential Predictions Using DFT Calculations

In the next step, the newly designed library was screened for identifying coumarin-like florescent probes against each heavy metal using the QSPR models. The screening results are presented in Table S18 (Supplementary Materials) in which the designed compounds are denoted as NEW, and the logβ and PS values of each were estimated using the respectively selected models. Of note, a potential probe must have a high logβ and a low PS, which in turn can also be translated into a high PS_ML_^−1^. Figure 4 shows the trade-off of the logβ and PS_ML_^−1^ predictions, in which compounds closer to the top-right corner indicate better candidates. According to this criterion, we easily identified eight compounds, *viz*. NEW02, NEW03, NEW07, NEW21, NEW26, NEW34, NEW51, and NEW80, as potential florescent probes. Their logβ and PS values estimated by all the QSPR models are shown in Table 4, and the chemical SMILES of these eight coumarins are listed in Table S19. Especially, compounds NEW02, NEW03, and NEW07 were consistently predicted to be good ligand candidates in complex with all three heavy metal ions.

**Figure 5:**
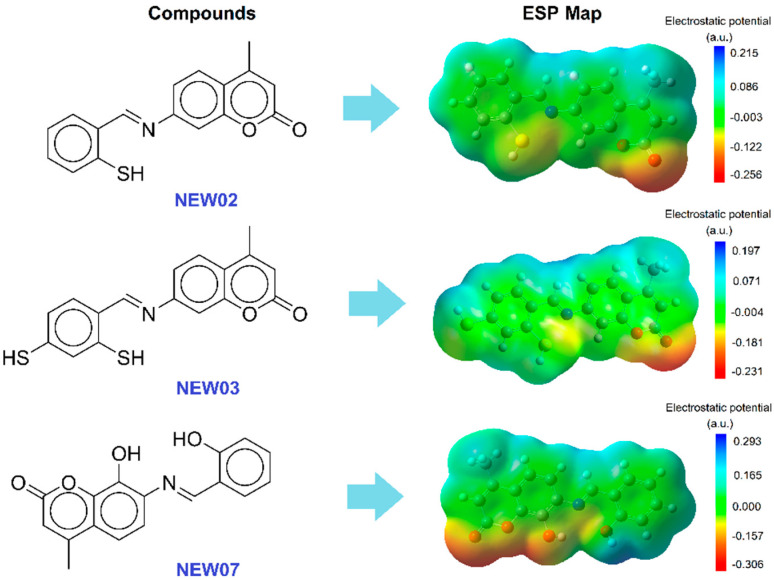
Structures of 3 novel coumarin-like compounds that have the potential to be heavy metal binders.

Resulting from the prediction of the complexing characteristics, three florescent probe candidates were revealed, and they were subsequently examined for their interaction with Cd^2+^, Cu^2+^, and Pb^2+^ using DFT calculations with B3LYP exchange functionals. To simplify the calculations, the stoichiometry of the complexes was adopted to be in a 1:1 (*M*:*L*) metal-and-coumarin-ligand molar ratio. In accordance with the literature, given the availability of a lone pair on the nitrogen atom, the Schiff base could form mono complexes with many metals; meanwhile, adding other substitutions, such as OH and SH, to the adjacent heterocycles may result in the formation of bidentate or tridentate chelates. This fact could be revealed by observing the partial distribution of the charge on the electrostatic potential surface (ESP) shown in Figure 5. This electrostatic potential map for all three compounds clearly revealed hydrophilic regions, including negative (red) and positive (blue) potentials, and hydrophobic regions, which are normally neutral (mainly the green area). The optimized geometric structures of the coumarin compounds computed via the B3LYP method showed that the reactive sites corresponding to the negative potentials were mainly localized on the O and S atoms, and two compounds, NEW02 and NEW07, showed higher polarity compared to NEW03. The total electron density of the studied derivatives varied from −0.31 to +0.29.

**Figure 6:**
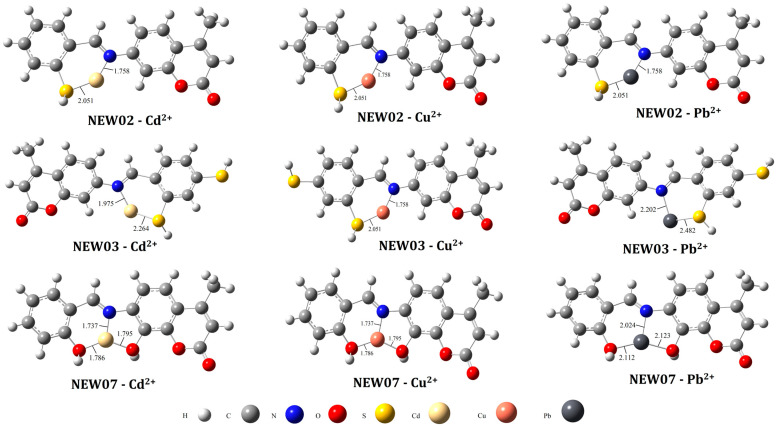
Optimized structures of 3 potential coumarin-like compounds in complex with 3 heavy metal ions.

The quantum chemical parameters can provide insight into the chemical nature of the reaction mechanisms [95]. Through the DFT calculations at the B3LYP/LanL2DZ levels of theory, numerous descriptors corresponding to six metal complexes were determined as summarized in Table 5. Figure 6 shows the optimized structures of the metal–coumarin complexes and the corresponding distances of the π contacts between the ligands and metals. The molecular frontier orbitals (HOMO–LUMO) consisted of important descriptors mainly associated with the molecular reactivity.

**Figure 7:**
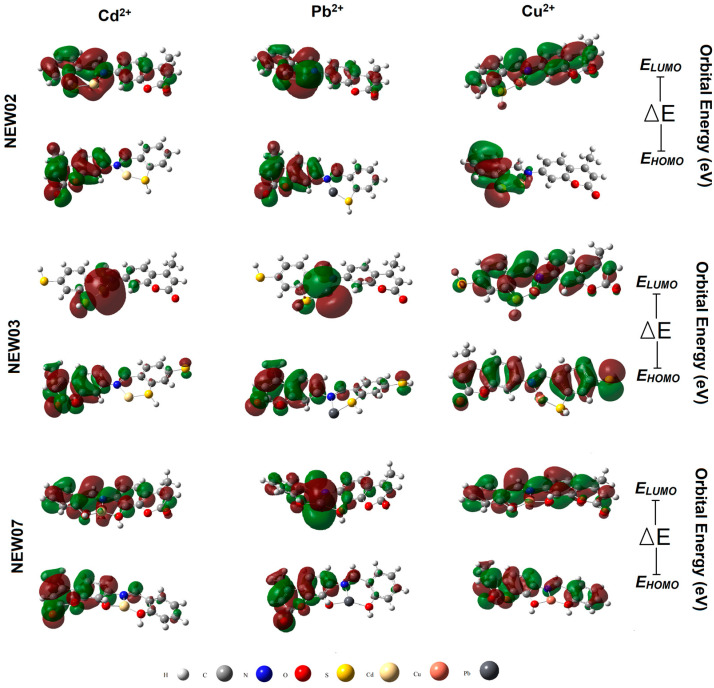
Frontier molecular orbitals of three complexes relevant to fluorescence quenching.

The E_HOMO_ descriptor is related to the ability of the molecules to donate electrons to empty molecular orbitals with low energy, and the E_LUMO_ descriptor indicates the electron-accepting affinity [96]. The interacting parts of the molecules were also identified by looking at the HOMO and LUMO visualization (Figure 7). Their different HOMO–LUMO ΔE represent the hard–soft, acid–base characteristics of the molecule. In such a way, the low gap energies identified in this study reflected the high chemical reactivity, optical polarizability, and low kinetic stability of the three metal complexes [97]. In other words, a molecule with a higher HOMO and a lower LUMO energy level has a higher chemical reactivity. Thus, for the Cd^2+^ complexes, the energy gap followed the order: NEW03 < NEW02 < NEW07 (see Table 5), indicating that NEW03 had the lowest reactivity (the most-chemically stable) followed by NEW03 and NEW07 (the most-reactive). Likewise, the HOMO–LUMO gap for the Cu^2+^ complexes were ranked as NEW03 < NEW07 < NEW02, and those for the Pb^2+^ complexes were similar to the Cd^2+^ complexes. In addition, the electron affinity (EA) and electronegativity (*χ*) of all six complexes were quite low compared to the ionization energy (IE). The current molecular orbital analysis also showed that the level of the frontier orbitals and low-energy electronic transitions were almost similar, suggesting the low energy intra-ligand charge transfer states of the metal complexes [95,97].

Another descriptor of pivotal interest is the chemical hardness, *η*, which refers to the resistance of a chemical species to the polarization of the electron cloud. It gives information, along with the electronegativity of the chemical reactivity and stability [79,95,96]. The softness is the reciprocal value of hardness. Previous studies demonstrated that most hard species have an electrostatic interaction, and soft species mainly interact through covalent bonding, e.g., mixing of orbitals. Of note, the global softness value increased from NEW07 to NEW03, while the chemical hardness and HOMO–LUMO gap values decreased. Evidently, the Schiff bases gained softer characteristics from NEW03 to NEW07. It was revealed that the stability parameters obtained in the QSPR predictions and the rankings obtained in the quantum chemical calculations were in concordance (Table 4 and Table 5). In addition, Cu^2+^ and Cd^2+^ had higher electron donor characteristics (*α*) compared to Pb^2+^, while Cu^2+^ and Pb^2+^ showed higher basicity characteristics (*β*) compared to Cd^2+^. The softness parameter *σ* can also be calculated via equation *σ* = *α*/(*α* + *β*), which follows the ranking Cd^2+^ > Cu^2+^ > Pb^2+^ (*σ* = 0.96 > 0.89 > 0.85, respectively). Thus, according to the Pearson classification for Lewis acids, Cu^2+^ and Pb^2+^ fall within the hard/soft borderline and Cd^2+^ is the softest ion. As soft acids prefer to bind to soft bases, compound NEW03, therefore, can easily interact with the softer Cd^2+^ charged ions, but not with the harder Pb^2+^ ion. As soft metals normally have a filled d subshell, according to Lancashire (https://chem.libretexts.org/, assessed on 3 March 2023), the π interactions play an important role in metal-to-ligand bonding. Complexes of three coumarins with softer metals are more stable based on the electrostatic interactions [95,97].

### 3.5. Physicochemical and Toxicological Profiling

Given the broad applications in the analytic field, a well-organized chemosensor must be non-toxic to living organisms when it is applied in aqueous environments [15]. Therefore, in this study, the toxicological profiles of the newly designed compounds were assessed using several cheminformatics tools, including SwissADME and ADMETlab 2.0 [81,82].

**Figure 8:**
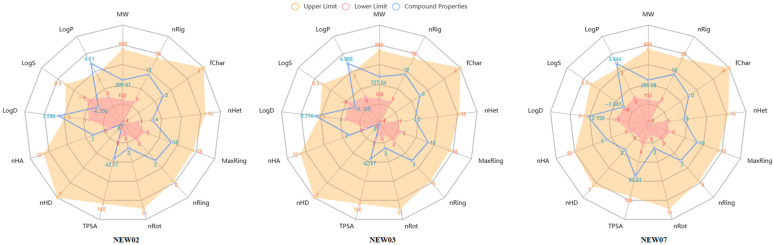
Physicochemical profiles of the 3 designed coumarin-like ligands. MW, molecular weight (kDa); log P, log of octanol partition coefficient; log D, log of octanol partition coefficient at physiological pH 7.4; fChar: formal charge; log S, log of aqueous solubility (mol/L); nHD, number of hydrogen bond donors; nHA, number of hydrogen bond acceptors; nHet, number of heteroatoms; nRot, number of rotatable bonds; nRing, number of rigid bonds; nRing, number of rings; MaxRing, number of atoms in the biggest ring; TPSA, topological polar surface area (Å^2^).

First, the physicochemical properties that may affect the interactions between chemicals and living organisms were computed for NEW02, NEW03, and NEW07. The radar chart shown in Figure 8 illustrates the multivariate data of all three coumarins compared to the physicochemical profiles of the drug-like chemical space [82]. Being small molecules (MW range from 295.3–327.5), most of the physicochemical properties of these compounds fell into the drug-like chemical space, except the lipophilicity indices (logP and logD). This means that these compounds may have a drug-like behavior including appropriate pharmacokinetic, as well as low toxicological characteristics in the human body [98]. Based on the consensus predictions of SwissADME (Table S20), the solubility of NEW02 and NEW03 was predicted to be low (logS < −6.0 mol/L), while NEW07 had higher solubility (logS = −5.4 mol/L). According to the combination of the three properties (MW, PSA, and logD_7.4_) as established in 3PRule [86], these coumarins may transport across the biological membrane via passive diffusion with moderate-to-high permeability. The oral bioavailability of these compounds was predicted to be <20%, and the brain disposition of these chemicals appeared to be low due to their low BBB passage [83].

Subsequently, the toxic effects on the human body were forecasted using both cheminformatics and toxicophore approaches. The results shown in Table 6 suggested that all three coumarins have no noticeable effects during exposure to the human body, especially on the skin. The oral acute toxicity was quite low, but for eye contact, they all can cause irritation [75,82]. In addition, biodegradability has been considered fundamental to the assessment of the environmental exposure and risk of chemical products. As can be seen in Table 6, there was no alert fragment in the structures of three coumarins, meaning that all the chemicals are biodegradable and will have no long-term toxic effect on the environment [82].

Finally, the ecotoxicological characteristics were assessed using cheminformatics tools implemented in ADMETlab 2.0. The results indicated that they had irrelevant environmental toxicity, especially for three types of aquatic organisms, e.g., *tetrahymena pyriformis* (TP), fathead minnow (FM), and *daphnia magna* (DM). These toxic endpoints are a mandatory preliminary assessment of chemicals’ acute toxicity on aquatic species, according to the European regulation on the Registration, Evaluation, Authorization and Restriction of Chemicals (REACH) [99]. As can be seen in Table 6, the lethal concentrations 50% (log unit) were determined to be from −4.5 to −6.6 mg/L, and NEW03 exhibited the lowest aquatic toxicity among the three compounds. The overall results obtained herein confirmed the appropriate organo-kinetic and biosafety level of the designed coumarins against human and environmental health, suggesting their suitability for industrial production as florescent chemosensors.

### 3.6. Theoretical Synthetic Routes Proposed for Designed Coumarins

Given the sensing characteristics’ predictions provided by the QSPR and quantum chemical calculations, it is desirable to obtain the florescent probes with the most potential for the experimental evaluations. Even though chemical synthesis was out of the scope of the current study, a proposal of the theoretical synthetic routes may be very helpful to assess the developability of the proposed structures, as well as to serve as a guidance for further developing novel chemosensors. To this end, three coumarins, NEW02, NEW03, and NEW07, were selected for the synthesis predictions.

The synthetic probability and SA scores of NEW02, NEW03, and NEW07 were first determined using the chemoinformatic tools GASA, ADMETlab, and SwissADME [81,82,87]. The results are summarized in Table 7, suggesting that all three chemicals belong to the easy-to-synthesize class.

**Scheme 1:**
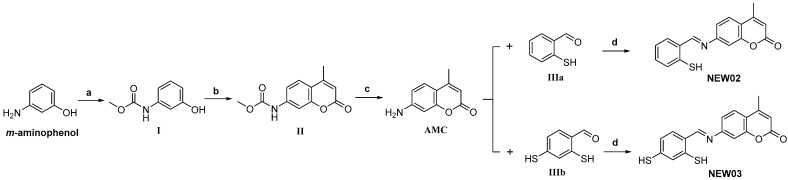
Reagents and conditions: (a) Methyl chloroformate, KHCO_3_, EtOAc, 4 h, r.t. (b) Ethyl 3-oxobutanoate, H_2_SO_4_ (70%), 18 h, r.t.. (c) H_2_SO_4_ (98%) and glacial acetic acid (1:1), reflux, 5 h. (d) EtOH, reflux, 10–12 h.

**Scheme 2:**
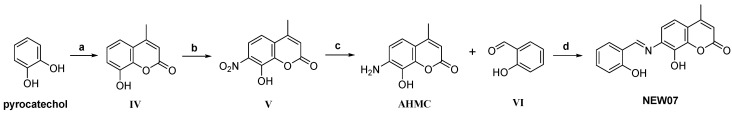
Reagents and conditions: (a) Ethyl 3-oxobutanoate, H_2_SO_4_ (70%), 24 h, 0 °C. (b) HNO_3_/H_2_SO_4_, DCM, 0 °C, 1 h. (**c**) ZnCl_2_, HCl, EtOAc, r.t. (d) NaBH_3_CN/MeOH, AcOH, r.t., 1 h.

Based on the synthetic accessibility predictions, we proposed in this study two theoretical synthetic routes: the first one for NEW02 and NEW03 and the other for NEW07. The first proposal, as depicted in Scheme 1, generates 7-amino-4-methyl coumarin (AMC) as a key intermediate, while the second one (Scheme 2) goes through another intermediate species, 7-amino-8-hydroxy-4-methylcoumarin (AHMC).

The theoretical synthetic routes proposed for NEW02 and NEW03 start with the preparation of methyl (3-hydroxyphenyl)carbamate (I) by acylation of the amino group in *m*-aminophenol with methyl chloroformate in the presence of a base (e.g., potassium bicarbonate). Subsequently, 7-carbethoxyamino-4-methylcoumarin (II) can be synthesized via Pechmann condensation with acetoacetic ester in sulfuric acid. Accordingly, the synthesis of AMC from II can be performed by heating II at reflux in H_2_SO_4_ (98%) and glacial acetic acid for around 5 h. After adjusting to a low temperature and to pH ~ 7, the precipitate is then dissolved in the ethanol solvent. The resulting ethanol solution can be used to obtain the target compounds (NEW02 or NEW03) by adding to other ethanol solutions containing 2-mercaptobenzaldehyde (IIIa) or 2,4-mercaptobenzaldehyde (IIIb), then the mixture must be refluxed for 10–12 h under stirring. Once finished, the end product is cooled to room temperature, and after that, it can be collected by filtration, dried, and purified by recrystallization from DMF and ethanol. Considering the previous reports [91], the intermediate AMC can be formed in good yield (~90%); thus, the recrystallized form of NEW02 and NEW03 can be produced in an overall yield greater than 50%.

The preparation of the remaining coumarin NEW07 based on the AHMC derivative is described by Scheme 2. The theoretical synthetic route is composed of four steps, starting with the formation of 8-hydroxy-4-methylcoumarin (IV) via a Pechmann condensation by reacting pyrocatechol and ethyl 3-oxobutanoate. Subsequently, nitration of IV with a mixture of nitric acid and sulfuric acid can lead to a separable mixture of both 7- and 8-nitrocoumarin derivatives. There are several methods proposed in the literature to reduce o-nitrohydroxy-4-methylcoumarins using different reducing agents [91,94]. In this study, 7-amino-8-hydroxy-4-methylcoumarin (AHMC) was obtained by the reduction of 8-hydroxy-4-methyl-7-nitrocoumarin (V) using zinc chloride in hydrochloric acid and ethyl acetate to achieve a better yield. Finally, the target compound NEW07 can be obtained by reductive amination of AHMC with 2-hydroxybenzaldehyde using sodium cyanoborohydride in methanol and acetic acid (Scheme 2).

## 4. Conclusions

Heavy metal contamination in wastewater from industrial activities is escalating at alarming levels, and it has now become a major global problem. There has been a great necessity to effectively develop analytical methods for the identification and quantification of heavy metals in the aquatic medium. Florescent chemosensors have attracted major attention of researchers in this field due to their significant advantages related to visual simplicity, low cost, high sensitivity, good selectivity, and rapid response compared to the other methods. In assisting the rational design of potential chemosensors, computational approaches, e.g., the QSPR and DFT, have been widely developed and applied for a variety of heavy metals over the last few decades. In this study, a number of QSPR models were developed using the GA-MLR statistical and 10 machine learning techniques on a large database of ~200 florescent chemosensor structures in complexes with three heavy metals (Cu^2+^, Cd^2+^, and Pb^2+^) previously reported. The modeling process clearly confirmed the ability of ML algorithms to improve the predictive performance of computational models. Six models based on GA-MLR, the AdaBoost regressor MLR, gradient boosting, and support vector regression, were selected and showed a good goodness-of-fit, robustness, and predictivity with the cross-validation accuracy ranging from >0.9 to 1.0, and those for external validation ranged from >0.8 to 0.95. It is worth noting that, so far, ML techniques have not been commonly used, then the predictive ability of the previously published studies appeared to be limited.

Subsequently, the developed computational models were applied for screening a virtual database of 129 coumarin-like structures, which were automatically generated based on seven potential florescent probes formerly reported in the literature. Interestingly, three novel simple compounds, namely NEW02, NEW03, and NEW07 were discovered with simultaneously high values of the stability constant and potentiometric sensitivity against three metal ions, Cu^2+^, Cd^2+^, and Pb^2+^. The QSPR predictions were then in good agreement with the quantum calculations using DFT/B3LYP approaches. All the HOMO–LUMO levels and other reactivity descriptors were in the same rankings as the QSPR approaches. To go further with the developability of the newly designed coumarin derivatives as chemosensors, their organo-kinetic and toxicological profiles were examined by computing numerous physicochemical and several toxic endpoints against human and environmental health using several cheminformatics tools. The results once again confirmed their safety characteristics for both humans and other species in aquatic ecosystems. Finally, based on retrosynthesis analysis with computational tools, the theoretical synthetic proposal for the three coumarins designed was successfully provided. The compounds can be synthesized by an inexpensive and simple method with good yield. Based on the aforementioned findings, the newly designed coumarins could be considered for further development stages as potential candidates as chemosensors against three metal ions, Cu^2+^, Cd^2+^, and Pb^2+^. The general workflow presented herein is promising, and we believe it can be applied to assist the development of novel chemosensors for screening multiple heavy metals’ contamination, which is one of the major environmental problems facing our planet today.

## Figures and Tables

**Table 1:** Summary of relevant reports of QSPR models for heavy metal chemosensor property prediction.

Year	Chemometric Methods	Metal Ions	Modeling Property	Chemical Structures
Kiani-Anbouhi et al. (2014) [31]	Genetic algorithm-multiple linear regression (GA-MLR); $R_{LOO}^{2}=0.86$; $R_{LGO}^{2}=0.86$; N = 29 ligands;	La^3+^	Stability constant (logβL)	Diverse
Kiani-Anbouhi et al. (2015) [34]	GA-MLR; $R_{LOO}^{2}=0.91$; $Q_{Test}^{2}=0.94$; N = 24 ligands;	Sm^3+^	Stability constant (logβL)	Diverse
Soloviev et al. (2019) [29]	Consensus MLR models; Cu^2+^: $Q_{Det}^{2}=0.64$; RMSE = 8.3; Zn^2+^: $Q_{Det}^{2}=0.78$; RMSE = 4.5; Cd^2+^: $Q_{Det}^{2}=0.77$; RMSE = 4.8; Pb^2+^: $Q_{Det}^{2}=0.76$; RMSE = 5.9; N = 35 ligands;	Cu^2+^, Zn^2+^, Cd^2+^, and Pb^2+^	Potentiometric sensitivity (PS_ML_)	Nitrogen-containing ligands (mainly diamides of pyridine and bipyridine acids)
Martynko et al. (2020) [33]	MLR models; $Q_{Det}^{2}=0.4$; RMSE = 8.8; N = 67 ligands; $Q_{Det}^{2}=0.66$; RMSE = 5.3; N = 56 ligands (refined data);	Mg^2+^/Ca^2+^	Selectivity coefficient logK(Mg^2+^/Ca^2+^)	Amide ionophores
Vladimirova et al. (2022) [30]	Partial least-squares regression (PLS); Cu^2+^: $Q_{Test}^{2}=0.66$; RMSE = 6.9; Cd^2+^: $Q_{Test}^{2}=0.81$; RMSE = 4.2; Pb^2+^: $Q_{Test}^{2}=0.64$; RMSE = 7.5; N = 35 ligands;	Cu^2+^, Cd^2+^, and Pb^2+^	Potentiometric sensitivity (PS_ML_)	Nitrogen-containing ligands (mainly diamides of pyridine and bipyridine acids)
Kanahashi et al. (2022) [35]	Gaussian process regression; Best model (8 cations, 49 ligands, 2 experimental conditions): MAE = 1.31; $R_{Training}^{2}=0.84$; N = 2706 ligands (*unpublished data*)	57 cations	Stability constant (logβL)	Diverse

**Table 2:** Overview of the performance parameters of the six MLR models.

	Statistical Parameters ^1^	MLR-logβ_CdL_	MLR-logβ_CuL_	MLR-logβ_PbL_	MLR-PS_CdL_	MLR-PS_CuL_	MLR-PS_PbL_
Fitting criteria	*K*_XX_	0.4121	0.2649	0.2384	0.4845	0.3958	0.2546
	*δK*	0.0698	0.0577	0.0679	0.0193	0.0679	0.0554
	*R* ^2^	0.9257	0.9085	0.8084	0.9160	0.8904	0.9264
	$R_{adj}^{2}$	0.9079	0.8862	0.7648	0.8957	0.8669	0.9080
	LOF	2.6866	7.0495	1.3524	2.4842	3.1127	3.7405
	*p*-value (F-test)	6.522 × 10^−13^	1.095 × 10^−19^	3.087 × 10^−7^	6.602 × 10^−14^	3.491 × 10^−12^	3.399 × 10^−14^
Internal validation criteria	$Q_{LOO}^{2}$	0.8637	0.8434	0.6918	0.8760	0.8423	0.8668
	*R*^2^ −$Q_{LOO}^{2}$	0.0620	0.0651	0.1165	0.0400	0.0481	0.0596
	$Q_{LMO}^{2}$	0.8311	0.8209	0.5872	0.8556	0.8246	0.8394
	*Y_scr_R* ^2^	0.1935	0.1975	0.1869	0.1938	0.1757	0.1961
	*Y_scr_Q* ^2^	−0.3624	−0.3143	−0.5988	−0.3579	−0.3038	−0.4169
External validation criteria	$Q_{F1}^{2}$	0.8324	0.8326	0.6357	0.8791	0.8914	0.7079
	$Q_{F2}^{2}$	0.8120	0.8276	0.6305	0.8789	0.8793	0.7054
	$Q_{F3}^{2}$	0.9283	0.8415	0.6961	0.9305	0.8080	0.7734

^1^ The statistical parameters correspond to the best MLR obtained based on the GA.

**Table 3:** Resulting hyperparameters for the best QSPR models selected for predicting the logβ and PS_ML_ data.

Data	Selected Model and Method	Optimized Parameter Settings ^1^
logβ (Cd^2+^ complex)	Model M1/AdaBoost Regressor MLR	Base_estimator=LinearRegression; copy_X=True, fit_intercept=True, n_jobs=None, normalize=‘deprecated’, positive=False); learning_rate=1.0; loss=‘linear’; n_estimators=50; random_state=None; fitting 5 folds for each of 100 candidates; totaling 500 fits {‘n_estimators’: 4, ‘loss’: ‘square’, ‘learning_rate’: 0.01}
PS_ML_ (Cd^2+^ complex)	Model M2/Gradient Boosting Regressor	Alpha=0.9; ccp_alpha=0.0; criterion=‘friedman_mse’; init=None; learning_rate=0.1; loss=‘squared_error’; max_depth=3; max_features=None; max_leaf_nodes=None; min_impurity_decrease=0.0; min_samples_leaf=1; min_samples_split=2; min_weight_fraction_leaf=0.0; n_estimators=100; n_iter_no_change=None; random_state=None; subsample=1.0, tol=0.0001; validation_fraction=0.1; verbose=0, warm_start=False {‘n_estimators’: 91, ‘min_samples_split’: 10, ‘min_samples_leaf’: 1, ‘max_features’: ‘sqrt’, ‘max_depth’: 6, ‘learning_rate’: 0.5}
logβ (Cu^2+^ complex)	Model M3/GA-MLR	Equation (11)
PS_ML_ (Cu^2+^ complex)	Model M4/Gradient Boosting Regressor	C=1.0, cache_size=200, coef0=0.0, degree=3, epsilon=0.1, gamma=‘scale’, kernel=‘rbf’, max_iter=−1, shrinking=True, tol=0.001, verbose=False {‘kernel’: ‘linear’, ‘gamma’: 10.0, ‘epsilon’: 0.5, ‘C’: 100.0}
logβ (Pb^2+^ complex)	Model M5/Gradient Boosting Regressor	Alpha=0.9; ccp_alpha=0.0; criterion=‘friedman_mse’; init=None, learning_rate=0.1, loss=‘squared_error’; max_depth=3; max_features=None; max_leaf_nodes=None; min_impurity_decrease=0.0; min_samples_leaf=1; min_samples_split=2; min_weight_fraction_leaf=0.0; n_estimators=100; n_iter_no_change=None; random_state=None; subsample=1.0; tol=0.0001; validation_fraction=0.1; verbose=0, warm_start=False {‘n_estimators’: 57, ‘min_samples_split’: 2, ‘min_samples_leaf’: 1, ‘max_features’: ‘sqrt’, ‘max_depth’: 8, ‘learning_rate’: 1}
PS_ML_ (Pb^2+^ complex)	Model M6/AdaBoost Regressor MLR	Base_estimator=LinearRegression; copy_X=True; fit_intercept=True; n_jobs=None; normalize=‘deprecated’; positive=False; learning_rate=1.0; loss=‘linear’; n_estimators=50; random_state=None; fitting 5 folds for each of 100 candidates; totaling 500 fits {‘n_estimators’: 42, ‘loss’: ‘linear’, ‘learning_rate’: 0.1}

^1^ The best hyperparameter combinations are shown within curly brackets.

**Table 4:** Overview of the performance parameters of the six machine learning models.

Compound ID	Stability Constant (logβ)			Potentiometric Sensitivity (PS_ML_, mV/dec)		
	Cd^2+^	Cu^2+^	Pb^2+^	Cd^2+^	Cu^2+^	Pb^2+^
NEW02	9.537	9.022	3.133	12.146	13.809	16.135
NEW03	10.263	9.610	2.935	14.901	16.194	16.947
NEW07	8.278	8.870	4.304	4.516	5.518	17.973
NEW21	6.912	5.506	4.024	4.746	22.307	16.812
NEW26	7.087	7.701	2.539	7.230	34.360	27.286
NEW34	6.550	6.607	3.435	4.236	28.975	23.507
NEW51	9.566	9.252	2.608	8.119	30.902	21.520

**Table 5:** The quantum chemical parameters of three coumarin compounds calculated at B3LYP/LanL2DZ levels.

Global Reactivity Index	DFT Energy (eV)								
	Cd^2+^ Complexes			Cu^2+^ Complexes			Pb^2+^ Complexes		
	NEW02	NEW03	NEW07	NEW02	NEW03	NEW07	NEW02	NEW03	NEW07
HOMO–LUMO Gap, ΔE	4.004	3.839	4.328	4.586	4.419	4.439	3.983	3.839	4.300
E_HOMO_	−6.074	−5.947	−6.305	−6.950	−6.774	−6.803	−6.225	−6.044	−6.410
E_LUMO_	−2.070	−2.108	−1.977	−2.363	−2.354	−2.364	−2.242	−2.205	−2.109
Ionization Energy, IE	6.074	5.947	6.305	6.950	6.774	6.803	6.225	6.044	6.410
Electron Affinity, EA	2.070	2.108	1.977	2.363	2.355	2.364	2.242	2.205	2.109
Global Hardness, *η*	2.002	1.919	2.164	2.293	2.210	2.219	1.992	1.920	2.150
Global Softness, *σ*	0.500	0.521	0.462	0.436	0.453	0.451	0.502	0.521	0.465
Electronegativity, *χ*	4.072	4.027	4.141	4.656	4.564	4.584	4.234	4.125	4.259
Electrophilicity, *ω*	4.142	4.225	3.962	4.727	4.715	4.734	4.500	4.431	4.219

**Table 6:** Relevant organo-kinetic and toxicological parameters of designed coumarins.

Parameters	NEW02	NEW03	NEW07	Description
Oral bioavailability (F_20%_)	0.982	0.999	0.968	The probability of oral bioavailability of 20% (F20%+). Category 1: F_20%_+ (bioavailability < 20%); Category 0: F_20%_− (bioavailability ≥ 20%).
Skin permeability	−5.47	−5.56	−6.39	logKp (cm/s).
Blood–brain barrier (BBB) penetration	0.039	0.107	0.023	The output value is the probability of being permeable (BBB+). Category 1: BBB+; Category 0: BBB−.
Acute oral toxicity	0.049	0.097	0.381	The output value is the probability of being highly toxic (Category 0: low–toxicity; Category 1: high–toxicity).
**Toxicophore predictions**				
Acute toxicity rule	0 alert	0 alert	0 alert	Predicted as toxic towards water sources based on 99 substructures
Non-biodegradable rule	0 alert	0 alert	0 alert	Predicted as non-biodegradable substances based on 19 substructures.
Skin sensitization rules	1 alert	1 alert	0 alert	Predicted as skin irritants based on 155 substructures.
Eye irritation	0.991	0.978	0.917	The output value is the probability of being irritants (Category 1: irritants; Category 0: non-irritants).
**Environmental toxicity**				
IGC_50_	−5.367	−5.796	−4.465	*Tetrahymena pyriformis* 50% growth inhibition concentration. The unit is log_10_[(mg/L)/(MW × 10^3^)].
LC_50_FM	−5.981	−6.466	−4.975	96 h fathead minnow 50% lethal concentration. The unit is log_10_[(mg/L)/(MW × 10^3^)].
LC_50_DM	−6.191	−6.547	−5.153	48 h *daphnia magna* 50% lethal concentration. The unit is log_10_[(mg/L)/(MW × 10^3^)].

**Table 7:** Synthetic accessibility prediction for selected coumarins.

Cpd. ID	Synthetic Probability ^1^	SwissADME SA Score ^2^	ADMETlab 2.0 SA Score ^3^	Consensus Classification
NEW02	0.879	3.14	2.62	ES (easy-to-synthesize)
NEW03	0.900	3.17	3.01	ES (easy-to-synthesize)
NEW07	0.899	3.36	2.63	ES (easy-to-synthesize)

Synthetic accessibility: ^1^ GASA synthetic probability ranges from 0 (hard-to-synthesize) to 1 (easy-to-synthesize); ^2^ SwissADME SA scores range from 1 (very easy) to 10 (very difficult); ^3^ ADMETlab 2.0 SA score classification: SA score ≥ 6, difficult-to-synthesize; SA score < 6, easy-to-synthesize.

## Data Availability

All the data for model development and application are provided.

## Supplementary Materials

The following Supporting Information can be downloaded at: https://www.mdpi.com/article/10.3390/toxics11070595/s1, Figure S1: Scatter plots of LMO validation approach; Figure S2: Y randomization test compared to the original QSPR model; Figures S3–S8: Correlation matrix of molecular descriptors selected in the six QSPR models; Table S1: Database of experimental potentiometric sensitivity (PS) of complexes between Cu^2+^, Cd^2+^, and Pb^2+^ ions and compounds; Tables S2–S4: Experimental stability constant (logβ) of the complex between three metals (Cd^2+^, Cu^2+^, Pb^2+^) towards compounds in the database; Tables S5–S10: Runtime parameters optimized for the ML algorithm predicting the logβ and PS_ML_ of the complex between three metals (Cd^2+^, Cu^2+^, Pb^2+^) and compounds in the database; Table S11: Molecular descriptors using in the GA-MLR models; Tables S12–S17: Performance comparison between the GA-MLR and ML models; Table S18: logβ and PS_ML_ parameter predictions for virtual the chemical library based on the corresponding QSPR models; Table S19: SMILES code of the 8 new coumarin-like structures and their leverage value (*h*_i_) computed by the 6 QSPR models; Tables S20–S26. Physicochemical and absorption, distribution, metabolism, and excretion (ADME) properties of three candidates based on the SwissADME tool.
