# Supplementary material for: Stability Constant and Potentiometric Sensitivity of Heavy Metal–Organic Fluorescent Compound Complexes: QSPR Models for Prediction and Design of Novel Coumarin-like Ligands
Source: Toxics. 2023 Jul 7;11(7):595. doi: 10.3390/toxics11070595 (PMC10383909; doi:10.3390/toxics11070595)
Supplement: Supplementary file 1 [file toxics-11-00595-s001.zip › toxics-2400301-supplementary.pdf]

Stability Constant and Potentiometric Sensitivity of Heavy Metal – Organic Fluorescent Compound Complexes: QSPR Models for Prediction and Design of Novel Coumarin–Like Ligands

Phan Thi Diem-Tran [1,†], Tue-Tam Ho [2,†], Nguyen-Van Tuan [2], Le-Quang Bao [2], Ha Tran Phuong [1], Trinh Thi Giao Chau [1], Hoang Thi Binh Minh [1], Cong-Truong Nguyen [2], Zulayho Smanova [3], Gerardo M. Casanola-Martin [4], Bakhtiyor Rasulev [3,4,*], Hai Pham-The [2,*] and Le Canh Viet Cuong [1,*]

[1] Mientrung Institute for Scientific Research, Vietnam National Museum of Nature, Vietnam Academy of Science and Technology, Hue 53000, Vietnam

[2] Faculty of Pharmaceutical Chemistry and Technology, Hanoi University of Pharmacy, 13-15 Le Thanh Tong, Hoan Kiem, Hanoi 10000, Vietnam

[3] Department of Chemistry, National University of Uzbekistan after Mirzo Ulugbek, Tashkent 100012, Uzbekistan

[4] Department of Coatings and Polymeric Materials, North Dakota State University, Fargo, ND 58102, USA

* Correspondence: bakhtiyor.rasulev@ndsu.edu (B.R.); hpham.phd@gmail.com (H.P.-T.); lcvcuong@vnmn.vast.vn (L.C.V.C.)

† These authors contributed equally to this work.

Supplementary materials:

Table S1. Database of experimental potentiometric sensitivity (PS) of complexes between Cu^{2+}, Cd^{2+}, Pb^{2+} ions and compounds

No.	SMILES	Code	PS_{ML} (mV/dec)		
			Cu^{2+}	Cd^{2+}	Pb^{2+}
1	O=C(COCC(N(CCCCCCCC)CCCCCCCC)=O)N(CCCCCCCC)CCCCCCCC	SEN01	5	9	30
2	O=C(C(C)OCC(N(CCCCCCCC)CCCCCCCC)=O)N(CCCCCCCC)CCCCCCCC	SEN02	12	13	24
3	O=C(C(C)OC(C)C(N(CCCCCCCC)CCCCCCCC)=O)N(CCCCCCCC)CCCCCC CC	SEN03	9	13	18
4	O=C(N(CC)CC)C1=CC=CC(C(N(CC)CC)=O)=N1	SEN04	25	14	27
5	O=C(N1CCCCC1)C2=CC=CC(C(N3CCCCC3)=O)=N2	SEN05	23	14	26
6	O=C(N(CC(C)C)CC(C)C)C1=CC=CC(C(N(CC(C)C)CC(C)C)=O)=N1	SEN06	34	27	51
7	O=C(N(C)C1CCCCC1)C2=NC(C(N(C)C3CCCCC3)=O)=CC=C2	SEN07	31	21	37
8	O=C(N(CCC1)C2=C1C=CC=C2)C3=NC(C(N(CCC4)C5=C4C=CC=C5)=O)=CC= C3	SEN08	34	26	34
9	O=C(N(CC)C1=C(C)C=CC=C1)C2=NC(C(N(CC)C3=CC=CC=C3C)=O)=CC=C2	SEN09	43	32	44
10	O=C(N(CC)C1=CC=C(C)C=C1)C2=NC(C(N(CC)C3=CC=C(C)C=C3)=O)=CC=C2	SEN10	43	32	45
11	O=C(N(CC)C1=CC=C(F)C=C1)C2=NC(C(N(CC)C3=CC=C(F)C=C3)=O)=CC=C2	SEN11	37	37	37
12	O=C(NCCOC1=CC=CC=C1)C2=NC(C(NCCOC3=CC=CC=C3)=O)=CC=C2	SEN12	28	23	28
13	O=C(NCCSCC)C1=NC(C(NCCSCC)=O)=CC=C1	SEN13	27	22	28
14	O=C(NCCOCCCCCCCCC)C1=NC(C(NCCOCCCCCCCCC)=O)=CC=C1	SEN14	31	24	31
15	O=C(NCCOC1=CC=CC=C1OC)C2=NC(C(NCCOC3=C(OC)C=CC=C3)=O)=CC= C2	SEN15	28	25	29
16	O=C(N(COC)CCOC1=CC=CC=C1)C2=NC(C(N(COC)CCOC3=CC=CC=C3)=O) =CC=C2	SEN16	25	22	42
17	O=C(N(C1=CC=CC=C1)CC)C2=CC=CC(C3=NC(C(N(C4=CC=CC=C4)CC)=O)= CC=C3)=N2	SEN17	31	36	24
18	O=C(N(CCCC)CCCC)C1=CC=CC(C2=NC(C(N(CCCC)CCCC)=O)=CC=C2)=N1	SEN18	39	37	26
19	O=C(N(C1=CC=CC=C1)CC)C2=CC(Br)=CC(C3=NC(C(N(C4=CC=CC=C4)CC)= O)=CC(Br)=C3)=N2	SEN19	26	36	28

20	O=C(N(C1=CC=C(F)C=C1)CC)C2=CC=CC(C3=NC(C(N(C4=CC=C(F)C=C4)CC)=O)=CC=C3)=N2	SEN20	30	31	23
21	O=C(N(C1=CC=C(CCCCCC)C=C1)CC)C2=CC=CC(C3=NC(C(N(C4=CC=C(CCCCCC)C=C4)CC)=O)=CC=C3)=N2	SEN21	34	36	23
22	O=C(N(C1=CC=C(CCCCCC)C=C1)CC)C2=CC(Br)=CC(C3=NC(C(N(C4=CC=C(CCCCCC)C=C4)CC)=O)=CC(Br)=C3)=N2	SEN22	31	41	27
23	O=C(N(C1=CC=CC=C1)CC)C2=CC=CC(C3=NC=CC=C3)=N2	SEN23	-	3	0
24	O=C(O)C1=CC=CC(C2=NC=CC=C2)=N1	SEN24	1	6	0
25	N#CC1=CC=CC(C2=NC=CC=C2)=N1	SEN25	-10	5	3
26	O=C1NNC(N1/N=C/C2=C(OCCOC3=CC=CC=C3/C=N/N4C(NNC4=O)=O)C=CC=C2)=O	SEN26	15	16	24
27	O=C1NNC(N1/N=C/C2=C(OCCOCCOC3=CC=CC=C3/C=N/N4C(NNC4=O)=O)C=CC=C2)=O	SEN27	15	15	24
28	SC1=NN=C(CC)N1/N=C/C2=C(OCCOC3=CC=CC=C3/C=N/N4C(CC)=NN=C4S)C=CC=C2	SEN28	19	17	25
29	SC1=NN=C(CC)N1/N=C/C2=C(OCCOCCOC3=CC=CC=C3/C=N/N4C(CC)=NN=C4S)C=CC=C2	SEN29	34	24	24
30	NC1=NN=CN1/N=C/C2=C(OCCOC3=CC=CC=C3/C=N/N4C(N)=NN=C4)C=CC=C2	SEN30	28	18	27
31	NC1=NN=CN1/N=C/C2=C(OCCOCCOC3=CC=CC=C3/C=N/N4C=NN=C4N)C=CC=C2	SEN31	20	18	28
32	O=C(N(CCCC)CCCC)C1=NC2=C3C(C=CC(C(N(CCCC)CCCC)=O)=N3)=CC=C2C=C1	SEN32	24	26	31
33	O=C(N(C1=CC=C(F)C=C1)CC)C2=NC3=C4C(C=CC(C(N(CC)C5=CC=C(F)C=C5)=O)=N4)=CC=C3C=C2	SEN33	23	27	26
34	O=C(N/N=C/C1=NC=CC=C1)C2=NC(C(N/N=C\C3=NC=CC=C3)=O)=CC=C2	SEN34	0	7	0
35	O=C(N/N=C/C1=C(O)C=CC=C1)C2=NC(C(N/N=C\C3=C(O)C=CC=C3)=O)=CC=C2	SEN35	0	5	4
36	O=P(CCCCCCCC)(CCCCCCCC)CCCCCCCC	SEN36	9	18	9
37	O=P(C1=CC=CC=C1)(C2=CC=CC=C2)C3=CC=CC=C3	SEN37	5	–10	–20
38	O=P(C1=CC=CC=C1)(C2=CC=CC=C2)CP(C3=CC=CC=C3)(C4=CC=CC=C4)=O	SEN38	26	23	38
39	O=P(C1=CC=CC=C1)(C2=CC=CC=C2)COCCOCCOCP(C3=CC=CC=C3)(C4=CC=CC=C4)=O	SEN39	30	25	33
40	O=P(C1=CC=CC=C1)(C2=CC=CC=C2)COCCOCCOCCOCCOCCOCP(C3=CC=CC=C3)(C4=CC=CC=C4)=O	SEN40	-	27	40
41	CP(C1=C(OCP(C2=CC=CC=C2)(C3=CC=CC=C3)=O)C=CC=C1)(C4=CC=CC=C4OCP(C5=CC=CC=C5)(C6=CC=CC=C6)=O)=O	SEN41	20	24	16
42	CCCN(CCN(CCC)CC(CP(C1=CC=CC=C1)(C2=CC=CC=C2)=O)=O)CC(CP(C3=CC=CC=C3)(C4=CC=CC=C4)=O)=O	SEN42	20.3	24.6	32.9
43	O=C(CP(C1=CC=CC=C1)(C2=CC=CC=C2)=O)CN(CCCCCCCC)CCCCN(CCCCCCCC)CC(CP(C3=CC=CC=C3)(C4=CC=CC=C4)=O)=O	SEN43	18.3	22.1	31.7
44	O=C(CP(C1=CC=CC=C1)(C2=CC=CC=C2)=O)CN(CCCCCCCC)CCCCCCN(CCCCCCCC)CC(CP(C3=CC=CC=C3)(C4=CC=CC=C4)=O)=O	SEN44	21.1	24.7	34.6
45	O=C(CP(C1=CC=CC=C1)(C2=CC=CC=C2)=O)N3CCN(C(CP(C4=CC=CC=C4)(C5=CC=CC=C5)=O)=O)CC3	SEN45	23.4	23	34.9

Table S2. Experimental stability constant (logβ) of complex between Cu^{2+} and compounds

No.	SMILES	Code	Logβ
1	CC(/C(C)=N/O)=N\NC(N(C1=CC=CC=C1)[H])=S	L1	6.468
2	CC(/C=N/NC1=CC=CC=C1)=N\NC(N(C)[H])=S	L2	12.14
3	S=C(N(C)C)N/N=C(/C(C1=CC=CC=C1)=N/O)C2=CC=CC=C2	L3	5.748
4	NC(N/N=C1C(N(C)C2=C\1C=CC=C2)=O)=S	L4	9.06
5	NC(N/N=C1CC=C(N)C=C\1)=S	L5	11.61
6	NC(N/N=C/C1=C(C=CC=C2)C2=CC=C1O)=S	L6	9.34

7	NC(N/N=C/C1=NC=CC=C1)=S	L7	20.4
8	NC(N/N=C/C1=CC=C(N(C)C)C=C1)=S	L8	15.3
9	NC(N/N=C/C1=C(O)C=CC=C1)=S	L10	19.1
10	NC(N/N=C/C1=CC=C(O)C=C1)=S	L11	17.2
11	NC(N/N=C/C1=CC=CC=C1)=S	L12	17.7
12	NC(N/N=C/C1=NC(C)=CC=C1)=S	L13	19.1
13	NC(N/N=C(C)/C1=CC=CC=N1)=S	L14	5.491
14	NC(N/N=C(C)/C1=CC=CN=C1)=S	L15	5.924
15	NC(N/N=C/C1=NC(C(O)=CC=C2)=C2C=C1)=S	L16	14.56
16	S=C(NCC)N/N=C/C1=NC(C(O)=CC=C2)=C2C=C1	L17	14.67
17	S=C(NC1=CC=CC=C1)N/N=C/C2=NC(C(O)=CC=C3)=C3C=C2	L18	15.65
18	NC(N/N=C/C1=CC(OC)=CC(OC)=C1O)=S	L19	6.236
19	NC(N/N=C(C)/C1=CC=CC=C1O)=S	L21	5.91
20	S=C(NC)N/N=C(C)/C1=CC=CC=N1	L22	6.114
21	S=C(N)N/N=C/C1=CC(C)=CC(CN2CCCC2C(O)=O)=C1O	L23	17.54
22	S=C(N)N/N=C/C1=CC=CC(OC)=C1O	L24	9.44
23	S=C(N(C)C)N/N=C(C1=NC=CC=C1)/C2=CC=CC=N2	L26	7.08
24	CC(/C=N/NC1=CC=CC=C1)=N\NC(N)=S	L27	11.95
25	O[C@@H]1CCCC[C@H]1NCCN[C@H]2[C@H](O)CCCC2	L28	11.47
26	O[C@@H]1CCCC[C@H]1NCCCN[C@H]2[C@H](O)CCCC2	L29	12.67
27	O[C@@H]1CCCC[C@H]1NCCNCCN[C@H]2[C@H](O)CCCC2	L30	16.74
28	OC(CN(CC(O)=O)CCN(CP(O)(O)=O)CCN(CC(O)=O)CC(O)=O)=O	L31	21.43
29	OC(CN(CC(O)=O)CCN(CP(O)(C1=CC=CC=C1)=O)CCN(CC(O)=O)CC(O)=O)=O	L32	19.47
30	CC(OP(O)(O)=O)=O	L33	2.86
31	CC(CP(O)(O)=O)=O	L34	3.36
32	OP(CCOCC)(O)=O	L35	3.44
33	OP(CCOCCN1C(N=CN=C2N)=C2N=C1)(O)=O	L36	3.98
34	CC1=NC(C2=NC(C3=NC=CC=C3)=CC=C2)=CC(C4=CC=CC(C5=CC=CC=N5)=N4)=N1	L37	12.6
35	NC1=NC=NC(C2=NC(C3=NC(C4=CC=CC=C4)=NC(C5=NC(C6=NC=NC(N)=C6)=CC=C5)=C3)=CC=C2)=C1	L38	14.4
36	CC(C1C2)(C)C2C(C1=C3)=NC=C3C4=CC=CC(C5=NC=C(C6=NC(C7=NC=C(C8C(C)(C)C9C8)C9=C7)=CC=C6)N=C5)=N4	L39	10
37	O=C(C1=CC=CC=C1)C2=NC=CC=C2	L40	4.287
38	CCN(CC)C1=CC=C2C(OC(C(C(NCC3=NC=CC=C3)=O)=C2)=O)=C1	L41	5.068
39	NCC(NC(CC1=CNC=N1)C(O)=O)=O	L42	8.68
40	NCC(NC(CC1=CNC=N1)C(NC([H])C(O)=O)=O)=O	L43	8.52
41	NC(CC1=CNC=N1)C(NCC(O)=O)=O	L44	8.02
42	N1(CC2=NC=CS2)CCN(CC3=CC=NS3)CCNCC1	L45	20.77
43	N1(CC2=NC=CS2)CCNCCCNCCNCCC1	L46	21.56
44	N1(CC2=NC=CS2)CCNCCCN(CC3=NC=CS3)CCNCCC1	L47	19.9
45	NC(CO)(CO)CO	L48	4.37
46	FC1=C(O)C=C(OC(C2=C3C4=CC=C(N(CC(O)=O)CC(O)=O)C(OC)=C4)=CC(C(F)=C2)=O)C3=C1	L50	6.009
47	O=C(CN(CC1C(OCOC)C(OCC2=CC=CC=C2)C(OCOC)C3O1)CCC4OC(CN(CC(NC5=CC=C6C7=C5C=CC8=C7C(CC=C8)=CC6)=O)CC3)C(OCOC)C(OCC9=CC=CC=C9)C4OCOC)NC(C=C%10)=C%11C=CC%12=CC=CC%13=CC=C%10C%11=C%12%13	L51	6.7
48	O=C(CN(CC1C(OC)C(OCC2=CC=CC=C2)C(OC)C3O1)CCC4OC(CN(CC(NC5=CC=C6C7=C5C=CC8=C7C(CC=C8)=CC6)=O)CC3)C(OC)C(OCC9=CC=CC=C9)C4OC)NC(C=C%10)=C%11C=CC%12=CC=CC%13=CC=C%10C%11=C%12%13	L52	7.8
49	NCC(O)=O	L53	8.15
50	O=C(O)CNCC(O)=O	L54	10.57
51	O=C(O)CN(CC(O)=O)CC(O)=O	L55	12.94
52	NCCNCC(O)=O	L56	13.47
53	O=C(O)CN(CC(O)=O)CCN(CC(O)=O)CC(O)=O	L57	18.7
54	NCCN	L58	10.54

55	NCCNCCNCCNCCN	L59	22.9
56	NC(CC(O)=O)C(O)=O	L60	9.08
57	NC(CC1=CC=CC=C1)C(O)=O	L61	7.53
58	NC(CC1=CNC2=C1C=CC=C2)C(O)=O	L62	8.47
59	NC(CCC(O)=O)C(O)=O	L63	8.28
60	NC(CC(N)=O)C(O)=O	L64	8.61
61	NC(CC(C)C)C(O)=O	L65	7.79
62	O=C(O)C1=CC=CC=C1	L66	8.4
63	O=C(O)COCCN(CC1)CCN1C(C2=CC=CC=C2)C3=CC=CC=C3	L67	9.12
64	CC(C)(C)C1=CC=C(C(NN(C)C)=O)C=C1	L68	4.17
65	CC(C)(C)C1=CC=C(C(NN(CCCC)CCCC)=O)C=C1	L69	3.6
66	C1=CN=CN1	L70	4.18
67	O=C(N)CCNCCNCCC(N)=O	L71	11.73
68	OC1=CC=CC=C1/C=N/C2=CC3=C(C(C)=CC(O3)=O)C=C2	L72	7.7
69	OC1=C(OC)C=CC=C1/C=N/C2=CC3=C(C(C)=CC(O3)=O)C=C2	L74	6.28
70	OC1=C(OCC)C=CC=C1/C=N/C2=CC3=C(C(C)=CC(O3)=O)C=C2	L75	6.3
71	OC1=CC(OC)=CC=C1/C=N/C2=CC3=C(C(C)=CC(O3)=O)C=C2	L76	8.29

Table S3. Experimental stability constant (logβ) of complex between Cd^{2+} and compounds

No.	SMILES	Code	Logβ
1	C1OCCNCCSCCNC1	L77	8.1
2	C1SCCOCCSCCNC1	L78	7.9
3	C1SCCSCCSCCNC1	L79	5.8
4	NCCCN1CCSCCOCCSCC1	L80	8.08
5	NCCCN1CCSCCSCCSCC1	L81	8.2
6	NCCCN1CCOCCN(CCCN)CCSCC1	L82	9.0
7	C1(CNCCNCCNC2)=NC(C(N=C2C=C3)=C3C=C4)=C4C=C1	L83	17.2
8	NCCN1CCNCC(C=C2)=NC3=C2C=CC4=C3N=C(CNCC1)C=C4	L84	18.83
9	C1(CSCCNCCSC2)=NC2=CC=C1	L85	9.12
10	NCCCN1CCSCC2=CC=CC(CSCC1)=N2	L86	10.3
11	C1CNCCNCCNCCS1	L87	9.91
12	C1(CN2C=C(C3=CC=CC=N3)C=N2)=CC=C(CN4N=C(C5=NC=CC=C5)C=C4)C=C1	L88	7.914
13	C1(CN2C=C(C3=CC=CC=N3)C=N2)=CC=CC(CN4C=CC(C5=CC=CC=N5)=N4)=C1	L89	7.064
14	C1(C2=NC=CC=C2)=NC=CC=C1	L90	4.5
15	BrC1=CC=C(/N=C/C2=C(O)C(O)=C(O)C=C2)C=C1	L91	6.67
16	OC1=C(C(C)(C)C)C=C(/N=C/C2=C(O)C(O)=C(O)C=C2)C=C1C(C)(C)C	L92	7.03
17	CC1=CC=C(/N=C/C2=C(O)C(O)=CC=C2)C=C1	L93	7.12
18	BrC1=CC=C(/N=C/C2=C(O)C(O)=CC=C2)C=C1	L94	7.09
19	OC1=C(C(C)(C)C)C=C(/N=C/C2=C(O)C=C(O)C=C2)C=C1C(C)(C)C	L95	6.86
20	NCC(O)=O	L53	4.26
21	NC(C(C)C)C(O)=O	L96	3.7
22	NC(CC(C)C)C(O)=O	L65	4.04
23	NC(C(CC)C)C(O)=O	L97	3.6
24	CCCC(N)C(O)=O	L98	3.73
25	CCCCC(N)C(O)=O	L99	3.86
26	NC(CC1=CC=CC=C1)C(O)=O	L61	3.6
27	NC(CC1=CNC2=C1C=CC=C2)C(O)=O	L62	4.51
28	NC(CCCNC(N)=N)C(O)=O	L100	3.27
29	NC(CC(O)=O)C(O)=O	L60	4.07
30	NC(CCC(N)=O)C(O)=O	L101	4.1
31	NC(CO)C(O)=O	L102	3.77
32	NC([C@@H](C)O)C(O)=O	L103	3.9
33	NC(CC1=CC=C(O)C=C1)C(O)=O	L104	3.56
34	NC(CC(O)=O)C(O)=O	L60	4.68

35	NC(CC1=CNC=N1)C(O)=O	L42	5.58
36	NC(CCSC)C(O)=O	L105	3.65
37	NC(CS)C(O)=O	L106	12.82
38	OC(C(S)CC(O)=O)=O	L107	10.05
39	NCCCC(N)C(O)=O	L108	3.41
40	NCCC1=CNC=N1	L109	4.78

Table S4. Experimental stability constant (logβ) of complex between Pb^{2+} and compounds

No.	SMILES	Code	Logβ
1	S=C1NN=C(C2=CC=CC=N2)C(C3=NC=CC=C3)(O)N1	L110	5.8
2	CC1(C2=CC=CS2)NC(C=CC=C3)=C3N=C(C4=CC=CS4)C1	L111	2.44
3	C12=CC=CC=C1C=C(OCCOCCOCCO3)C3=C2	L112	2.29
4	NC(N/N=C\C(CC(O)C(CO)O)=N/NC(N)=S)=S	L113	2.74
5	OC1(C2(O)C(O)CCCC2)CCCCC1O	L114	4.09
6	O=C(N/N=C\C1=CC=CO1)C2=CC=CO2	L115	2.9
7	CN1C(C)=C(NC(NC2=C(OC)C=CC=C2)=S)C(N1C3=CC=CC=C3)=O	L116	2.23
8	OC1C(O)(C2CCCC/C2=N/O)CCCC1	L117	2.45
9	C/C(C1=CC=CS1)=N\C2=C(N)C=CC=C2	L118	2.44
10	CC1=C(C(N)=O)C=NN1C2=CC=CC=C2	L119	2.74
11	NN(C(NN)=NN=C1C)C1=O	L120	2.73
12	C1(/C=N/CCC/N=C/C2=CC=CS2)=CC=CS1	L121	3.35
13	NN(C(NN=C1C)=S)C1=S	L122	2.23
14	O=C(NNS(=O)(C1=C(C=CC=C2N(C)C)C2=CC=C1)=O)NC3=CC=CC=C3	L123	2.1
15	OC(C1=CC=CC=C1)/C(C2=CC=CC=C2)=N/NC(C3=CC=CC=C3)=O	L124	2.74
16	CC(/C(C)=N/N=C\C1=CC=CS1)=N\N=C/C2=CC=CS2	L125	2.24
17	CC1=C(/N=C\N(C)/C=N/C2=CC=C(C)C=C2C)C=CC(C)=C1	L126	2.31
18	OC1=C(CC2=C(OC(C3=CC=CC=C3)=O)C(CC4=C(OC(C5=CC=CC=C5)=O)C6=CC(C(C)(C)C)=C4)=CC(C(C)(C)C)=C2)C=C(C(C)(C)C)C=C1CC7=CC(C(C)(C)C)=CC(C6)=C7OC(C8=CC=CC=C8)=O	L127	2.86
19	C1(N=C(/N=C\C2=CC=CS2)S3)=C3C=CC=C1	L128	2.2
20	S=C1SC(SCC2=CC=CC(CS3)=N2)=C(SCC4=CC=CC(CSC(S5)=C3SC5=S)=N4)S1	L129	2.1
21	O=C(N/N=C\C1=NC=CC=C1)C2=CC=CO2	L130	2.42
22	CC(/C(C)=N\N=C\C1=CC=C([N+]([O-])=O)O1)=N/N=C/C2=CC=C([N+]([O-])=O)O2	L131	2.95
23	O=C(NCCNC(C1=NC=CC=C1)=O)C2=CC=CC=N2	L132	2.74
24	S=C(N/N=C(C(C1=CC=CC=C1)=O)/C2=CC=CC=C2)NC3=CC=CC=C3	L133	2.9
25	CC1=C(C)C=C(NC(C2=NC(C=CC=C3)=C3C=C2)=O)C(NC(C4=CC=C(C=CC=C5)C5=N4)=O)=C1	L134	3.44
26	NC(N/N=C(C1=CC=CC=C1)/C(C2=CC=CC=C2)=N/NC(N)=S)=S	L135	4.42
27	N#CCN1CCOCCOCCN(CC#N)CCOCCOCC1	L136	6.9
28	O=[N+]([O-])C1=C(C(O)=O)C=C(SSC2=CC=C([N+]([O-])=O)C(C(O)=O)=C2)C=C1	L137	6.22
29	OC(C=CC=C1)=C1/C(C2=CC=CC=C2)=N/CCCN(CCC/N=C(C3=CC=CC=C3O)\C4=CC=CC=C4)CCC/N=C(C5=CC=CC=C5)/C6=C(O)C=CC=C6	L138	2.86
30	CC1=C(N=C(N/N=C/C2=CC=CS2)S3)C3=CC=C1	L139	2.74
31	O=C1C2=CC=CC3=CC=CC(C1=NNC(C4=CC=CO4)=O)=C32	L140	2.27
32	NC(N/N=C/C1=C(C=CC=C2)C2=CC=C1O)=S	L6	7.23
33	NC(N/N=C1C(C=CC=C2)=C2N(C)C\1=O)=S	L141	8.109
34	NC(N/N=C/C1=CC=CC(OC)=C1O)=S	L142	6.83
35	C/C(C1=CC=CC=C1O)=N\NC(N)=S	L143	5.01

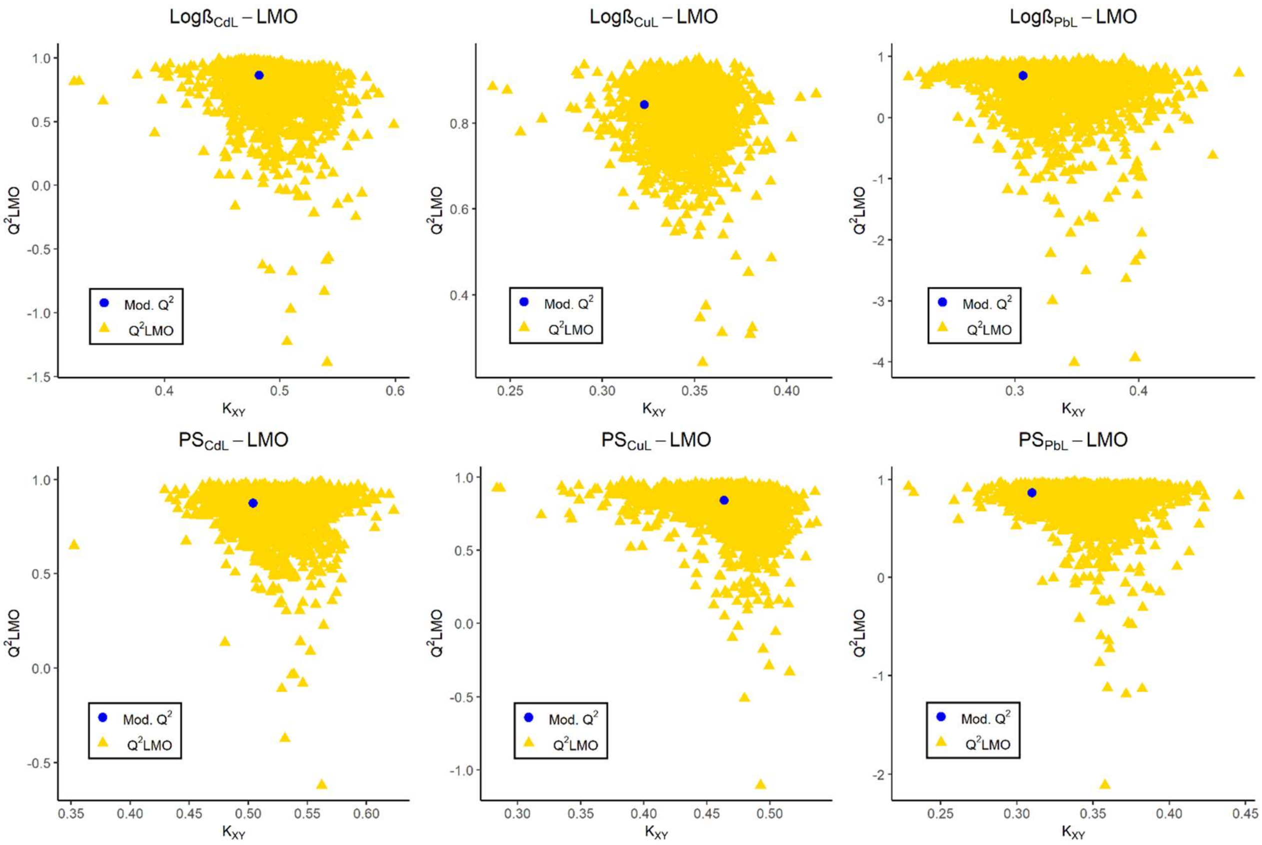

Figure S1. Scatter plots of LMO validation approach.

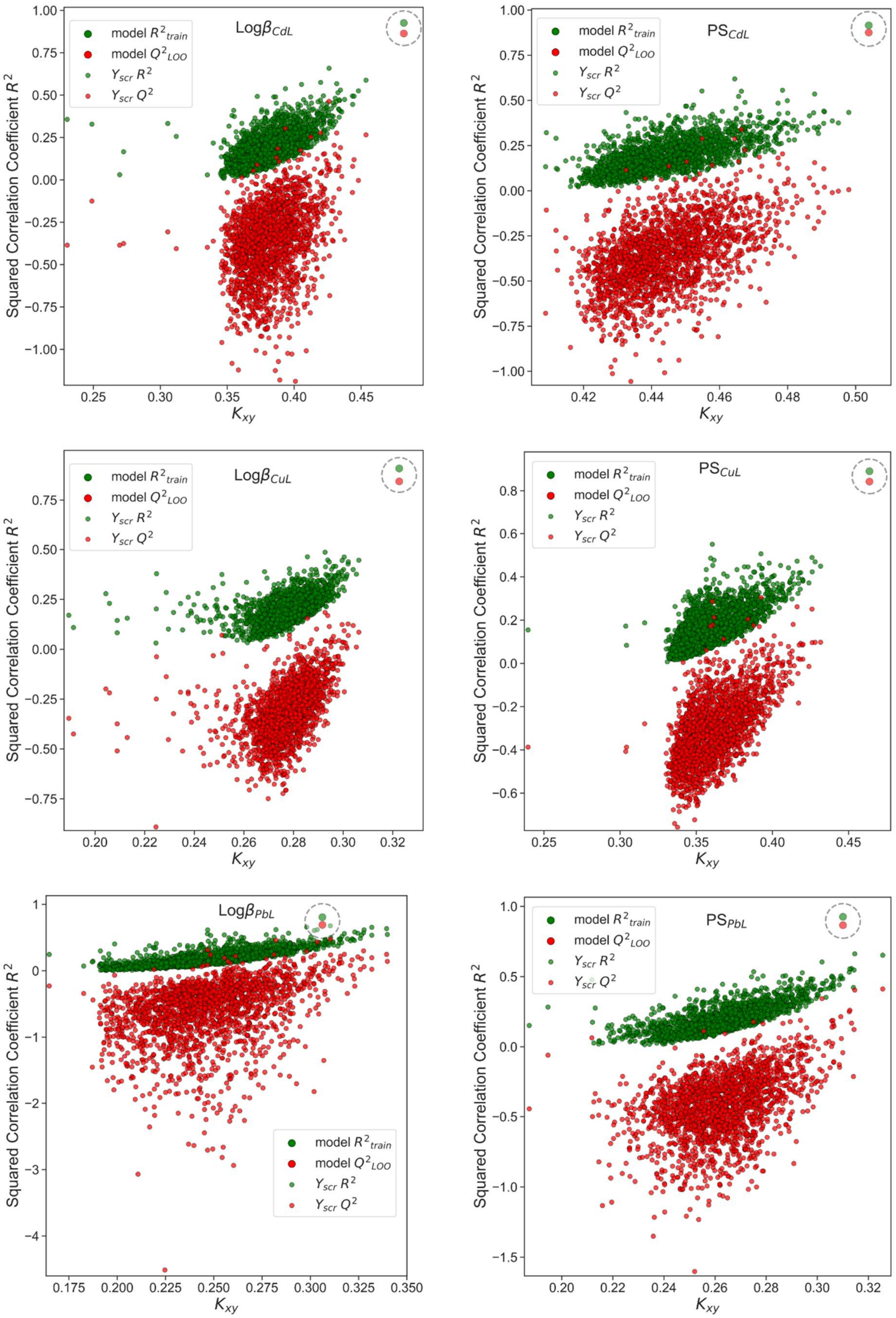

Figure S2. Y-Randomization test compared to original QSPR model.

Table S5. Runtime parameters optimized for ML algorithm predicting logβ of complex between Cd^{2+} and compounds.

Algorithms	Optimized Parameters
AdaBoostRegressor_DecisionTree Regressor	AdaBoostRegressor(base_estimator=DecisionTreeRegressor(ccp_alpha=0.0, criterion='squared_error', max_depth=6, max_features=None, max_leaf_nodes=None, min_impurity_decrease=0.0, min_samples_leaf=1, min_samples_split=2, min_weight_fraction_leaf=0.0, random_state=None, splitter='best'), learning_rate=1.0, loss='linear', n_estimators=50, random_state=None) {'n_estimators': 4, 'loss': 'square', 'learning_rate': 0.01}
AdaBoostRegressor_LinearRegres sion	AdaBoostRegressor(base_estimator=LinearRegression(copy_X=True, fit_intercept=True, n_jobs=None, normalize='deprecated', positive=False), learning_rate=1.0, loss='linear', n_estimators=50, random_state=None) Fitting 5 folds for each of 100 candidates, totalling 500 fits {'n_estimators': 4, 'loss': 'square', 'learning_rate': 0.01}
DecisionTreeRegressor	best hyperparameters {'splitter': 'random', 'min_weight_fraction_leaf': 0.1, 'min_samples_leaf': 2, 'max_leaf_nodes': 41, 'max_features': None, 'max_depth': 62}
GaussianProcessRegressor	GaussianProcessRegressor(alpha=1e-10, copy_X_train=True, kernel=None, n_restarts_optimizer=0, normalize_y=False, optimizer='fmin_l_bfgs_b', random_state=None) {'kernel': DotProduct(sigma_0=100), 'alpha': 0.1}
GradientBoostingRegressor	GradientBoostingRegressor(alpha=0.9, ccp_alpha=0.0, criterion='friedman_mse', init=None, learning_rate=0.1, loss='squared_error', max_depth=3, max_features=None, max_leaf_nodes=None, min_impurity_decrease=0.0, min_samples_leaf=1, min_samples_split=2, min_weight_fraction_leaf=0.0, n_estimators=100, n_iter_no_change=None, random_state=None, subsample=1.0, tol=0.0001, validation_fraction=0.1, verbose=0, warm_start=False) {'n_estimators': 36, 'min_samples_split': 10, 'min_samples_leaf': 1, 'max_features': 'log2', 'max_depth': 7, 'learning_rate': 1}
KNeighborsRegressor	KNeighborsRegressor(algorithm='auto', leaf_size=30, metric='minkowski', metric_params=None, n_jobs=None, n_neighbors=5, p=2, weights='uniform') {'weights': 'uniform', 'p': 1, 'n_neighbors': 4, 'metric': 'minkowski', 'leaf_size': 36}
MLPRegressor	MLPRegressor(activation='relu', alpha=0.0001, batch_size='auto', beta_1=0.9, beta_2=0.999, early_stopping=False, epsilon=1e-08, hidden_layer_sizes=(100,), learning_rate='constant', learning_rate_init=0.001, max_fun=15000, max_iter=100000, momentum=0.9, n_iter_no_change=10, nesterovs_momentum=True, power_t=0.5, random_state=None, shuffle=True, solver='adam', tol=0.0001, validation_fraction=0.1, verbose=False, warm_start=False) best hyperparameters {'solver': 'lbfgs', 'learning_rate': 'invscaling', 'hidden_layer_sizes': 43, 'activation': 'logistic'}
RandomForestRegressor	RandomForestRegressor(bootstrap=True, ccp_alpha=0.0, criterion='squared_error', max_depth=None, max_features=1.0, max_leaf_nodes=None, max_samples=None, min_impurity_decrease=0.0,

	min_samples_leaf=1, min_samples_split=2, min_weight_fraction_leaf=0.0, n_estimators=100, n_jobs=None, oob_score=False, random_state=None, verbose=0, warm_start=False) {'n_estimators': 4, 'min_samples_split': 2, 'min_samples_leaf': 2, 'max_features': 'log2', 'max_depth': 6, 'bootstrap': True}
SupportVectorRegressor	SVR(C=1.0, cache_size=200, coef0=0.0, degree=3, epsilon=0.1, gamma='scale', kernel='rbf', max_iter=-1, shrinking=True, tol=0.001, verbose=False) best hyperparameters {'kernel': 'linear', 'gamma': 1e-05, 'epsilon': 0.4, 'C': 0.001}

Table S6. Runtime parameters optimized for ML algorithm predicting PS_{ML} of complex between Cd^{2+} and compounds.

Algorithms	Optimized Parameters
AdaBoostRegressor	AdaBoostRegressor(base_estimator=DecisionTreeRegressor(ccp_alpha=0.0, criterion='squared_error', max_depth=6, max_features=None, max_leaf_nodes=None, min_impurity_decrease=0.0, min_samples_leaf=1, min_samples_split=2, min_weight_fraction_leaf=0.0, random_state=None, splitter='best'), learning_rate=1.0, loss='linear', n_estimators=50, random_state=None) {'n_estimators': 48, 'loss': 'linear', 'learning_rate': 0.5}
AdaBoostRegressor_MLR	AdaBoostRegressor(base_estimator=LinearRegression(copy_X=True, fit_intercept=True, n_jobs=None, normalize='deprecated', positive=False), learning_rate=1.0, loss='linear', n_estimators=50, random_state=None) Fitting 5 folds for each of 100 candidates, totalling 500 fits {'n_estimators': 73, 'loss': 'exponential', 'learning_rate': 0.01}
DecisionTreeRegressor	best hyperparameters {'splitter': 'best', 'min_weight_fraction_leaf': 0.3, 'min_samples_leaf': 11, 'max_leaf_nodes': 124, 'max_features': 'log2', 'max_depth': 47}
GaussianProcessRegressor	GaussianProcessRegressor(alpha=1e-10, copy_X_train=True, kernel=None, n_restarts_optimizer=0, normalize_y=False, optimizer='fmin_l_bfgs_b', random_state=None) {'kernel': DotProduct(sigma_0=10), 'alpha': 0.0001}
GradientBoostingRegressor	GradientBoostingRegressor(alpha=0.9, ccp_alpha=0.0, criterion='friedman_mse', init=None, learning_rate=0.1, loss='squared_error', max_depth=3, max_features=None, max_leaf_nodes=None, min_impurity_decrease=0.0, min_samples_leaf=1, min_samples_split=2, min_weight_fraction_leaf=0.0, n_estimators=100, n_iter_no_change=None, random_state=None, subsample=1.0, tol=0.0001, validation_fraction=0.1, verbose=0, warm_start=False) {'n_estimators': 91, 'min_samples_split': 10, 'min_samples_leaf': 1, 'max_features': 'sqrt', 'max_depth': 6, 'learning_rate': 0.5}
KNeighborsRegressor	KNeighborsRegressor(algorithm='auto', leaf_size=30, metric='minkowski', metric_params=None, n_jobs=None, n_neighbors=5, p=2, weights='uniform') {'weights': 'distance', 'p': 1, 'n_neighbors': 23, 'metric': 'minkowski', 'leaf_size': 37}

RandomForestRegressor	MLPRegressor(activation='relu', alpha=0.0001, batch_size='auto', beta_1=0.9, beta_2=0.999, early_stopping=False, epsilon=1e-08, hidden_layer_sizes=(100,), learning_rate='constant', learning_rate_init=0.001, max_fun=15000, max_iter=100000, momentum=0.9, n_iter_no_change=10, nesterovs_momentum=True, power_t=0.5, random_state=None, shuffle=True, solver='adam', tol=0.0001, validation_fraction=0.1, verbose=False, warm_start=False)<br>best hyperparameters {'solver': 'lbfgs', 'learning_rate': 'invscaling', 'hidden_layer_sizes': 14, 'activation': 'identity'}
RandomForestRegressor	RandomForestRegressor(bootstrap=True, ccp_alpha=0.0, criterion='squared_error', max_depth=None, max_features=1.0, max_leaf_nodes=None, max_samples=None, min_impurity_decrease=0.0, min_samples_leaf=1, min_samples_split=2, min_weight_fraction_leaf=0.0, n_estimators=100, n_jobs=None, oob_score=False, random_state=None, verbose=0, warm_start=False)<br>{'n_estimators': 89, 'min_samples_split': 2, 'min_samples_leaf': 1, 'max_features': 'sqrt', 'max_depth': 10, 'bootstrap': False}
SupportVectorRegressor	SVR(C=1.0, cache_size=200, coef0=0.0, degree=3, epsilon=0.1, gamma='scale', kernel='rbf', max_iter=-1, shrinking=True, tol=0.001, verbose=False)<br>best hyperparameters {'kernel': 'linear', 'gamma': 0.1, 'epsilon': 1, 'C': 100.0}

Table S7. Runtime parameters optimized for ML algorithm predicting logβ of complex between Cu^{2+} and compounds.

Algorithms	Optimized Parameters
AdaBoostRegressor	AdaBoostRegressor(base_estimator=DecisionTreeRegressor(ccp_alpha=0.0, criterion='squared_error', max_depth=6, max_features=None, max_leaf_nodes=None, min_impurity_decrease=0.0, min_samples_leaf=1, min_samples_split=2, min_weight_fraction_leaf=0.0, random_state=None, splitter='best'), learning_rate=1.0, loss='linear', n_estimators=50, random_state=None)<br>{'n_estimators': 5, 'loss': 'linear', 'learning_rate': 1}
AdaBoostRegressor	AdaBoostRegressor(base_estimator=LinearRegression(copy_X=True, fit_intercept=True, n_jobs=None, normalize='deprecated', positive=False), learning_rate=1.0, loss='linear', n_estimators=50, random_state=None)<br>Fitting 5 folds for each of 100 candidates, totalling 500 fits<br>{'n_estimators': 62, 'loss': 'linear', 'learning_rate': 0.1}
DecisionTreeRegressor	best hyperparameters {'splitter': 'random', 'min_weight_fraction_leaf': 0.1, 'min_samples_leaf': 4, 'max_leaf_nodes': 145, 'max_features': None, 'max_depth': 146}
GaussianProcessRegressor	GaussianProcessRegressor(alpha=1e-10, copy_X_train=True, kernel=None, n_restarts_optimizer=0, normalize_y=False, optimizer='fmin_l_bfgs_b', random_state=None)<br>{'kernel': DotProduct(sigma_0=10), 'alpha': 0.001}

GradientBoostingRegressor	GradientBoostingRegressor(alpha=0.9, ccp_alpha=0.0, criterion='friedman_mse',<br>init=None, learning_rate=0.1, loss='squared_error',<br>max_depth=3, max_features=None, max_leaf_nodes=None,<br>min_impurity_decrease=0.0, min_samples_leaf=1,<br>min_samples_split=2, min_weight_fraction_leaf=0.0,<br>n_estimators=100, n_iter_no_change=None,<br>random_state=None, subsample=1.0, tol=0.0001,<br>validation_fraction=0.1, verbose=0, warm_start=False)<br>{'n_estimators': 70, 'min_samples_split': 10, 'min_samples_leaf': 1, 'max_features':<br>'log2', 'max_depth': None, 'learning_rate': 0.5}
KNeighborsRegressor	KNeighborsRegressor(algorithm='auto', leaf_size=30, metric='minkowski',<br>metric_params=None, n_jobs=None, n_neighbors=5, p=2,<br>weights='uniform')<br>{'weights': 'distance', 'p': 2, 'n_neighbors': 2, 'metric': 'euclidean', 'leaf_size': 6}
MLPRegressor	MLPRegressor(activation='relu', alpha=0.0001, batch_size='auto', beta_1=0.9,<br>beta_2=0.999, early_stopping=False, epsilon=1e-08,<br>hidden_layer_sizes=(100,), learning_rate='constant',<br>learning_rate_init=0.001, max_fun=15000, max_iter=100000,<br>momentum=0.9, n_iter_no_change=10, nesterovs_momentum=True,<br>power_t=0.5, random_state=None, shuffle=True, solver='adam',<br>tol=0.0001, validation_fraction=0.1, verbose=False,<br>warm_start=False)<br>best hyperparameters {'solver': 'lbfgs', 'learning_rate': 'constant',<br>'hidden_layer_sizes': 84, 'activation': 'identity'}
RandomForestRegressor	RandomForestRegressor(bootstrap=True, ccp_alpha=0.0, criterion='squared_error',<br>max_depth=None, max_features=1.0, max_leaf_nodes=None,<br>max_samples=None, min_impurity_decrease=0.0,<br>min_samples_leaf=1, min_samples_split=2,<br>min_weight_fraction_leaf=0.0, n_estimators=100,<br>n_jobs=None, oob_score=False, random_state=None,<br>verbose=0, warm_start=False)<br>{'n_estimators': 4, 'min_samples_split': 5, 'min_samples_leaf': 4, 'max_features':<br>'log2', 'max_depth': 8, 'bootstrap': True}
SupporVectorRegressor	SVR(C=1.0, cache_size=200, coef0=0.0, degree=3, epsilon=0.1, gamma='scale',<br>kernel='rbf', max_iter=-1, shrinking=True, tol=0.001, verbose=False)<br>best hyperparameters {'kernel': 'linear', 'gamma': 0.1, 'epsilon': 1, 'C': 100.0}

Table S8. Runtime parameters optimized for ML algorithm predicting PS_{ML} of complex between Cu^{2+} and compounds.

Algorithms	Optimized Parameters
AdaBoostRegressor_DTR	AdaBoostRegressor(base_estimator=DecisionTreeRegressor(ccp_alpha=0.0,<br>criterion='squared_error',<br>max_depth=6,<br>max_features=None,<br>max_leaf_nodes=None,<br>min_impurity_decrease=0.0,<br>min_samples_leaf=1,<br>min_samples_split=2,<br>min_weight_fraction_leaf=0.0,<br>random_state=None,<br>splitter='best'),<br>learning_rate=1.0, loss='linear', n_estimators=50,<br>random_state=None)<br>{'n_estimators': 5, 'loss': 'linear', 'learning_rate': 0.5}
AdaBoostRegressor_MLR	AdaBoostRegressor(base_estimator=LinearRegression(copy_X=True,<br>fit_intercept=True, n_jobs=None, normalize='deprecated',<br>positive=False),

	learning_rate=1.0, loss='linear', n_estimators=50, random_state=None) Fitting 5 folds for each of 100 candidates, totalling 500 fits {'n_estimators': 78, 'loss': 'linear', 'learning_rate': 1}
DecisionTreeRegressor	best hyperparameters {'splitter': 'random', 'min_weight_fraction_leaf': 0.1, 'min_samples_leaf': 2, 'max_leaf_nodes': 13, 'max_features': 'log2', 'max_depth': 4}
GaussianProcessRegressor	GaussianProcessRegressor(alpha=1e-10, copy_X_train=True, kernel=None, n_restarts_optimizer=0, normalize_y=False, optimizer='fmin_l_bfgs_b', random_state=None) {'kernel': DotProduct(sigma_0=0.1), 'alpha': 0.0001}
GradientBoostingRegressor	GradientBoostingRegressor(alpha=0.9, ccp_alpha=0.0, criterion='friedman_mse', init=None, learning_rate=0.1, loss='squared_error', max_depth=3, max_features=None, max_leaf_nodes=None, min_impurity_decrease=0.0, min_samples_leaf=1, min_samples_split=2, min_weight_fraction_leaf=0.0, n_estimators=100, n_iter_no_change=None, random_state=None, subsample=1.0, tol=0.0001, validation_fraction=0.1, verbose=0, warm_start=False) {'n_estimators': 100, 'min_samples_split': 5, 'min_samples_leaf': 2, 'max_features': 'sqrt', 'max_depth': 6, 'learning_rate': 0.5}
KNeighborsRegressor	KNeighborsRegressor(algorithm='auto', leaf_size=30, metric='minkowski', metric_params=None, n_jobs=None, n_neighbors=5, p=2, weights='uniform') {'weights': 'uniform', 'p': 1, 'n_neighbors': 12, 'metric': 'manhattan', 'leaf_size': 12}
MLPRegressor	MLPRegressor(activation='relu', alpha=0.0001, batch_size='auto', beta_1=0.9, beta_2=0.999, early_stopping=False, epsilon=1e-08, hidden_layer_sizes=(100,), learning_rate='constant', learning_rate_init=0.001, max_fun=15000, max_iter=100000, momentum=0.9, n_iter_no_change=10, nesterovs_momentum=True, power_t=0.5, random_state=None, shuffle=True, solver='adam', tol=0.0001, validation_fraction=0.1, verbose=False, warm_start=False) best hyperparameters {'solver': 'lbfgs', 'learning_rate': 'constant', 'hidden_layer_sizes': 44, 'activation': 'identity'}
RandomForestRegressor	RandomForestRegressor(bootstrap=True, ccp_alpha=0.0, criterion='squared_error', max_depth=None, max_features=1.0, max_leaf_nodes=None, max_samples=None, min_impurity_decrease=0.0, min_samples_leaf=1, min_samples_split=2, min_weight_fraction_leaf=0.0, n_estimators=100, n_jobs=None, oob_score=False, random_state=None, verbose=0, warm_start=False) {'n_estimators': 8, 'min_samples_split': 5, 'min_samples_leaf': 4, 'max_features': 'sqrt', 'max_depth': 6, 'bootstrap': False}
SuportVectorRegressor	SVR(C=1.0, cache_size=200, coef0=0.0, degree=3, epsilon=0.1, gamma='scale', kernel='rbf', max_iter=-1, shrinking=True, tol=0.001, verbose=False) best hyperparameters {'kernel': 'linear', 'gamma': 10.0, 'epsilon': 0.5, 'C': 100.0}

Table S9. Runtime parameters optimized for ML algorithm predicting logβ of complex between Pb^{2+} and compounds.

Algorithms	Optimized Parameters
AdaBoostRegressor_DTR	AdaBoostRegressor(base_estimator=DecisionTreeRegressor(ccp_alpha=0.0, criterion='squared_error', max_depth=6, max_features=None, max_leaf_nodes=None, min_impurity_decrease=0.0,

	min_samples_leaf=1,<br>min_samples_split=2,<br>min_weight_fraction_leaf=0.0,<br>random_state=None,<br>splitter='best'),<br>learning_rate=1.0, loss='linear', n_estimators=50,<br>random_state=None)<br>{'n_estimators': 38, 'loss': 'square', 'learning_rate': 1}
AdaBoostRegressor_MLR	AdaBoostRegressor(base_estimator=LinearRegression(copy_X=True,<br>fit_intercept=True, n_jobs=None,<br>normalize='deprecated',<br>positive=False),<br>learning_rate=1.0, loss='linear', n_estimators=50,<br>random_state=None)<br>Fitting 5 folds for each of 100 candidates, totalling 500 fits<br>{'n_estimators': 13, 'loss': 'linear', 'learning_rate': 0.05}
DecisionTreeRegressor	best hyperparameters {'splitter': 'best', 'min_weight_fraction_leaf': 0.2,<br>'min_samples_leaf': 2, 'max_leaf_nodes': 194, 'max_features': 'sqrt', 'max_depth': 93}
GaussianProcessRegressor	GaussianProcessRegressor(alpha=1e-10, copy_X_train=True, kernel=None,<br>n_restarts_optimizer=0, normalize_y=False,<br>optimizer='fmin_l_bfgs_b', random_state=None)<br>{'kernel': DotProduct(sigma_0=0.1), 'alpha': 1}
GradientBoostingRegressor	GradientBoostingRegressor(alpha=0.9, ccp_alpha=0.0, criterion='friedman_mse',<br>init=None, learning_rate=0.1, loss='squared_error',<br>max_depth=3, max_features=None, max_leaf_nodes=None,<br>min_impurity_decrease=0.0, min_samples_leaf=1,<br>min_samples_split=2, min_weight_fraction_leaf=0.0,<br>n_estimators=100, n_iter_no_change=None,<br>random_state=None, subsample=1.0, tol=0.0001,<br>validation_fraction=0.1, verbose=0, warm_start=False)<br>{'n_estimators': 57, 'min_samples_split': 2, 'min_samples_leaf': 1, 'max_features':<br>'sqrt', 'max_depth': 8, 'learning_rate': 1}
KNeighborsRegressor	KNeighborsRegressor(algorithm='auto', leaf_size=30, metric='minkowski',<br>metric_params=None, n_jobs=None, n_neighbors=5, p=2,<br>weights='uniform')<br>{'weights': 'distance', 'p': 2, 'n_neighbors': 21, 'metric': 'manhattan', 'leaf_size': 8}
MLPRegressor	MLPRegressor(activation='relu', alpha=0.0001, batch_size='auto', beta_1=0.9,<br>beta_2=0.999, early_stopping=False, epsilon=1e-08,<br>hidden_layer_sizes=(100,), learning_rate='constant',<br>learning_rate_init=0.001, max_fun=15000, max_iter=100000,<br>momentum=0.9, n_iter_no_change=10, nesterovs_momentum=True,<br>power_t=0.5, random_state=None, shuffle=True, solver='adam',<br>tol=0.0001, validation_fraction=0.1, verbose=False,<br>warm_start=False)<br>best hyperparameters {'solver': 'lbfgs', 'learning_rate': 'invscaling',<br>'hidden_layer_sizes': 3, 'activation': 'tanh'}
RandomForestRegressor	RandomForestRegressor(bootstrap=True, ccp_alpha=0.0, criterion='squared_error',<br>max_depth=None, max_features=1.0, max_leaf_nodes=None,<br>max_samples=None, min_impurity_decrease=0.0,<br>min_samples_leaf=1, min_samples_split=2,<br>min_weight_fraction_leaf=0.0, n_estimators=100,<br>n_jobs=None, oob_score=False, random_state=None,<br>verbose=0, warm_start=False)<br>{'n_estimators': 2, 'min_samples_split': 10, 'min_samples_leaf': 2, 'max_features':<br>'log2', 'max_depth': 5, 'bootstrap': True}
SupportVectorRegressor	SVR(C=1.0, cache_size=200, coef0=0.0, degree=3, epsilon=0.1, gamma='scale',<br>kernel='rbf', max_iter=-1, shrinking=True, tol=0.001, verbose=False)<br>best hyperparameters {'kernel': 'sigmoid', 'gamma': 0.0001, 'epsilon': 0.01, 'C': 0.0001}

Table S10. Runtime parameters optimized for ML algorithm predicting PS_{ML} of complex between Pb^{2+} and compounds.

Algorithms	Optimized Parameters
AdaBoostRegressor_DTR	AdaBoostRegressor(base_estimator=DecisionTreeRegressor(ccp_alpha=0.0,<br>criterion='squared_error',<br>max_depth=6,<br>max_features=None,<br>max_leaf_nodes=None,<br>min_impurity_decrease=0.0,<br>min_samples_leaf=1,<br>min_samples_split=2,<br>min_weight_fraction_leaf=0.0,<br>random_state=None,<br>splitter='best'),<br>learning_rate=1.0, loss='linear', n_estimators=50,<br>random_state=None)<br>{'n_estimators': 8, 'loss': 'linear', 'learning_rate': 0.1}
AdaBoostRegressor_MLR	AdaBoostRegressor(base_estimator=LinearRegression(copy_X=True,<br>fit_intercept=True,<br>n_jobs=None, normalize='deprecated', positive=False),<br>learning_rate=1.0, loss='linear', n_estimators=50,<br>random_state=None)<br>Fitting 5 folds for each of 100 candidates, totalling 500 fits<br>{'n_estimators': 42, 'loss': 'linear', 'learning_rate': 0.1}
DecisionTreeRegressor	best hyperparameters {'splitter': 'best', 'min_weight_fraction_leaf': 0.1,<br>'min_samples_leaf': 2, 'max_leaf_nodes': 49, 'max_features': 'log2', 'max_depth': 177}
GaussianProcessRegressor	GaussianProcessRegressor(alpha=1e-10, copy_X_train=True, kernel=None,<br>n_restarts_optimizer=0, normalize_y=False,<br>optimizer='fmin_l_bfgs_b', random_state=None)<br>{'kernel': DotProduct(sigma_0=1), 'alpha': 0.0001}
GradientBoostingRegressor	GradientBoostingRegressor(alpha=0.9, ccp_alpha=0.0, criterion='friedman_mse',<br>init=None, learning_rate=0.1, loss='squared_error',<br>max_depth=3, max_features=None, max_leaf_nodes=None,<br>min_impurity_decrease=0.0, min_samples_leaf=1,<br>min_samples_split=2, min_weight_fraction_leaf=0.0,<br>n_estimators=100, n_iter_no_change=None,<br>random_state=None, subsample=1.0, tol=0.0001,<br>validation_fraction=0.1, verbose=0, warm_start=False)<br>{'n_estimators': 20, 'min_samples_split': 2, 'min_samples_leaf': 2, 'max_features':<br>'sqrt', 'max_depth': 6, 'learning_rate': 0.5}
KNeighborsRegressor	KNeighborsRegressor(algorithm='auto', leaf_size=30, metric='minkowski',<br>metric_params=None, n_jobs=None, n_neighbors=5, p=2,<br>weights='uniform')<br>{'weights': 'distance', 'p': 2, 'n_neighbors': 4, 'metric': 'euclidean', 'leaf_size': 22}
MLPRegressor	MLPRegressor(activation='relu', alpha=0.0001, batch_size='auto', beta_1=0.9,<br>beta_2=0.999, early_stopping=False, epsilon=1e-08,<br>hidden_layer_sizes=(100,), learning_rate='constant',<br>learning_rate_init=0.001, max_fun=15000, max_iter=100000,<br>momentum=0.9, n_iter_no_change=10, nesterovs_momentum=True,<br>power_t=0.5, random_state=None, shuffle=True, solver='adam',<br>tol=0.0001, validation_fraction=0.1, verbose=False,<br>warm_start=False)<br>best hyperparameters {'solver': 'adam', 'learning_rate': 'constant',<br>'hidden_layer_sizes': 51, 'activation': 'identity'}
RandomForestRegressor	RandomForestRegressor(bootstrap=True, ccp_alpha=0.0, criterion='squared_error',<br>max_depth=None, max_features=1.0, max_leaf_nodes=None,

	max_samples=None, min_impurity_decrease=0.0, min_samples_leaf=1, min_samples_split=2, min_weight_fraction_leaf=0.0, n_estimators=100, n_jobs=None, oob_score=False, random_state=None, verbose=0, warm_start=False) {'n_estimators': 26, 'min_samples_split': 2, 'min_samples_leaf': 2, 'max_features': 'log2', 'max_depth': 5, 'bootstrap': True}
SupporVectorRegressor	SVR(C=1.0, cache_size=200, coef0=0.0, degree=3, epsilon=0.1, gamma='scale', kernel='rbf', max_iter=-1, shrinking=True, tol=0.001, verbose=False) best hyperparameters {'kernel': 'rbf', 'gamma': 0.1, 'epsilon': 0.6, 'C': 100.0}

Table S11. Molecular descriptors using in the GA-MLR models.

Code	Description	Block
RBN	number of rotatable bonds	Constitutional indices
SPI	superpendentic index	Topological indices
H_D/Dt	Harary-like index from distance/detour matrix	2D matrix-based descriptors
MATS1m	Moran autocorrelation of lag 1 weighted by mass	2D autocorrelations
MATS2m	Moran autocorrelation of lag 2 weighted by mass	2D autocorrelations
MATS6i	Moran autocorrelation of lag 6 weighted by ionization potential	2D autocorrelations
nHM	number of heavy atoms	Constitutional indices
ATSC4e	Centred Broto-Moreau autocorrelation of lag 4 weighted by Sanderson electronegativity	2D autocorrelations
MATS1v	Moran autocorrelation of lag 1 weighted by van der Waals volume	2D autocorrelations
MATS6p	Moran autocorrelation of lag 6 weighted by polarizability	2D autocorrelations
MATS7p	Moran autocorrelation of lag 7 weighted by polarizability	2D autocorrelations
GATS5p	Geary autocorrelation of lag 5 weighted by polarizability	2D autocorrelations
P_VSA_MR_7	P_VSA-like on Molar Refractivity, bin 7	P_VSA-like descriptors
SM07_AEA(ed)	spectral moment of order 7 from augmented edge adjacency mat. weighted by edge degree	Edge adjacency indices
O-057	phenol / enol / carboxyl OH	Atom-centered fragments
NssCH2	Number of atoms of type ssCH2	Atom-type E-state indices
B06[C-O]	Presence/absence of C - O at topological distance 6	2D Atom Pairs
nR05	number of 5-membered rings	Ring descriptors
J_Dt	Balaban-like index from detour matrix	2D matrix-based descriptors
ATSC1s	Centred Broto-Moreau autocorrelation of lag 1 weighted by I-state	2D autocorrelations
GATS8m	Geary autocorrelation of lag 8 weighted by mass	2D autocorrelations
SM03_EA(bo)	spectral moment of order 3 from edge adjacency mat. weighted by bond order	Edge adjacency indices
J_B(s)	Balaban-like index from Burden matrix weighted by I-State	2D matrix-based descriptors
GATS8i	Geary autocorrelation of lag 8 weighted by ionization potential	2D autocorrelations
JGI8	mean topological charge index of order 8	2D autocorrelations
SpMax4_Bh(m)	largest eigenvalue n. 4 of Burden matrix weighted by mass	Burden eigenvalues
SpMin3_Bh(s)	smallest eigenvalue n. 3 of Burden matrix weighted by I-state	Burden eigenvalues
SpMin5_Bh(s)	smallest eigenvalue n. 5 of Burden matrix weighted by I-state	Burden eigenvalues
DLS_01	modified drug-like score from Lipinski (4 rules)	Drug-like indices
Ho_Dz(p)	Hosoya-like index (log function) from Barysz matrix weighted by polarizability	2D matrix-based descriptors
GGI10	topological charge index of order 10	2D autocorrelations
P_VSA_p_2	P_VSA-like on polarizability, bin 2	P_VSA-like descriptors

Chi1_EA(dm)	connectivity-like index of order 1 from edge adjacency mat. weighted by dipole moment	Edge adjacency indices
DLS_04	modified drug-like score from Chen et al. (7 rules)	Drug-like indices
LLS_01	modified lead-like score from Congreve et al. (6 rules)	Drug-like indices
MATS6v	Moran autocorrelation of lag 6 weighted by van der Waals volume	2D autocorrelations
MATS2i	Moran autocorrelation of lag 2 weighted by ionization potential	2D autocorrelations
GATS4i	Geary autocorrelation of lag 4 weighted by ionization potential	2D autocorrelations
GGI8	topological charge index of order 8	2D autocorrelations
Eta_F_A	eta average functionality index	ETA indices

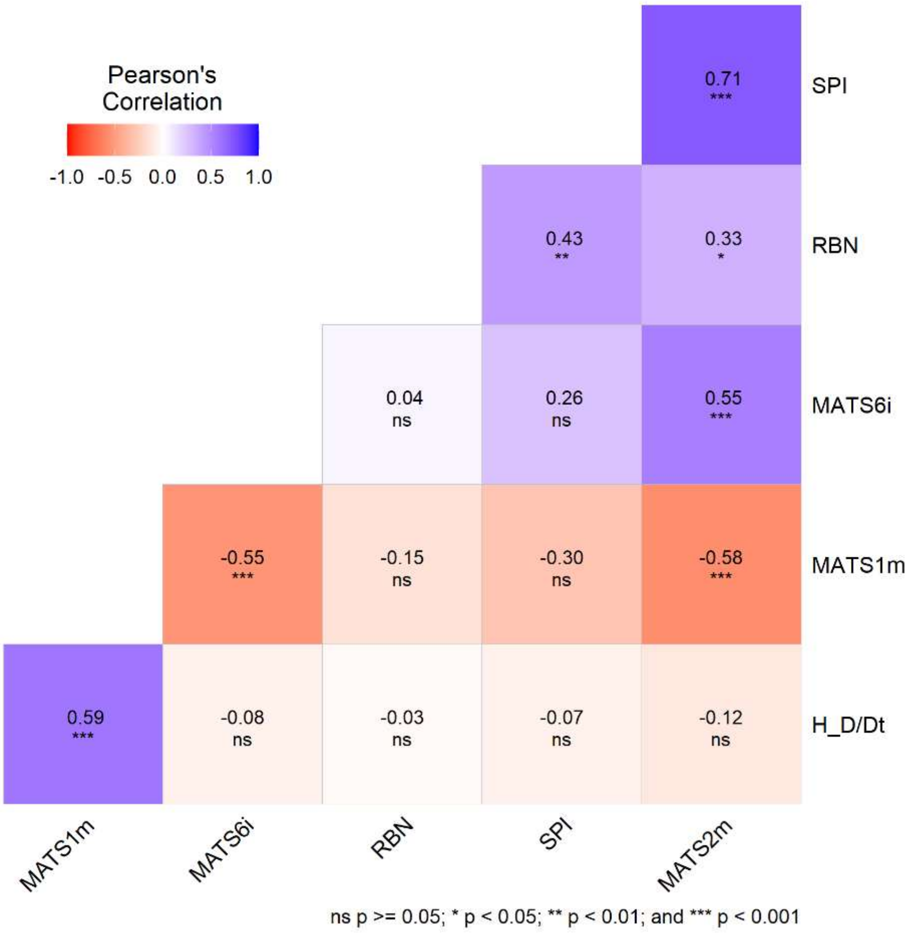

Figure S3. Correlation matrix of molecular descriptors selected in Cd^{2+} logβ QSPR model.

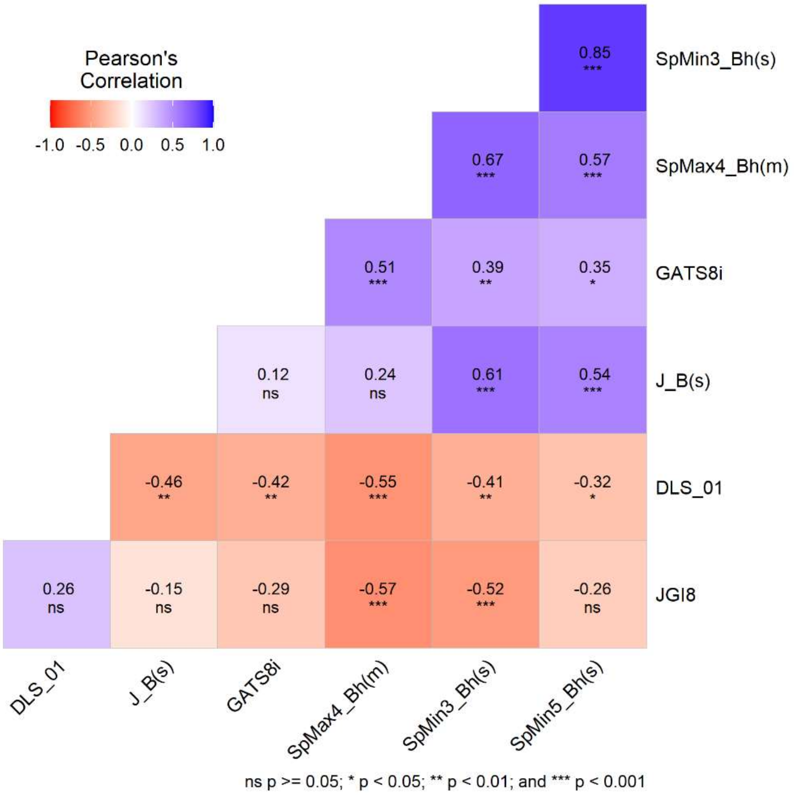

Figure S4. Correlation matrix of molecular descriptors selected in Cd^{2+} PS_{ML} QSPR model.

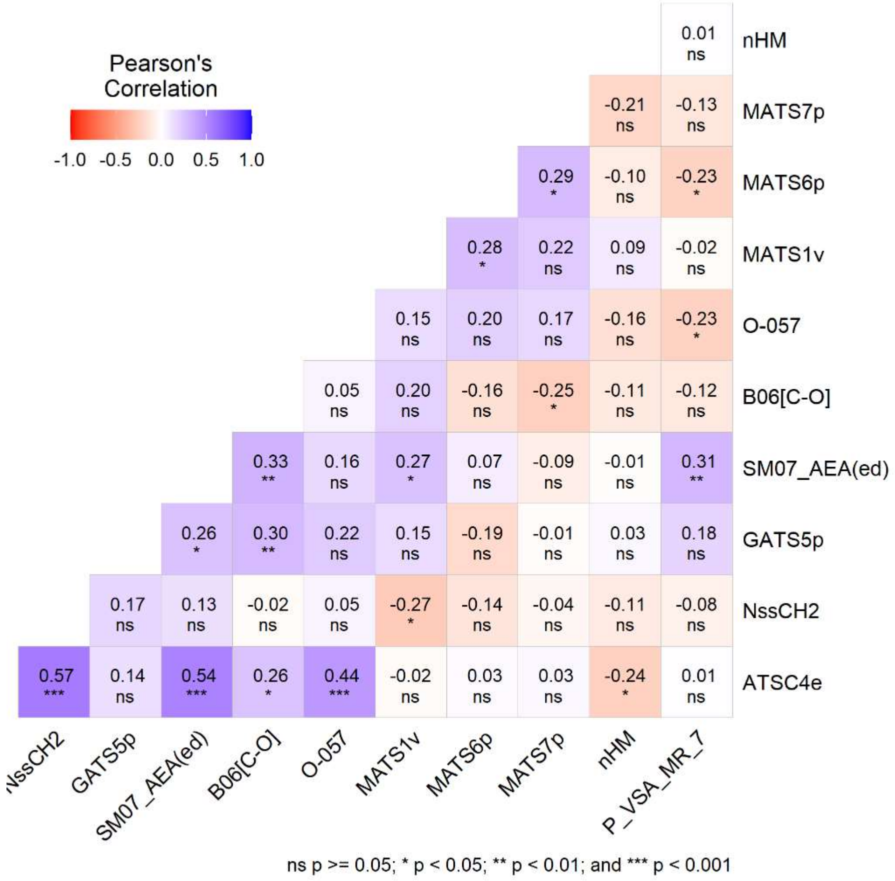

Figure S5. Correlation matrix of molecular descriptors selected in Cu^{2+} $\log\beta$ QSPR model.

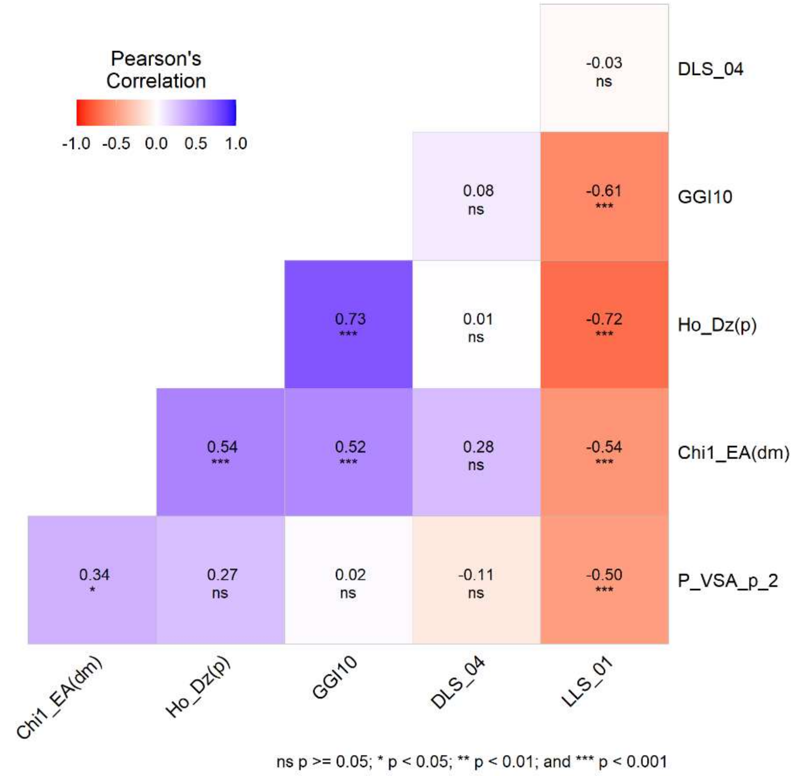

Figure S6. Correlation matrix of molecular descriptors selected in Cu^{2+} PS_{ML} QSPR model.

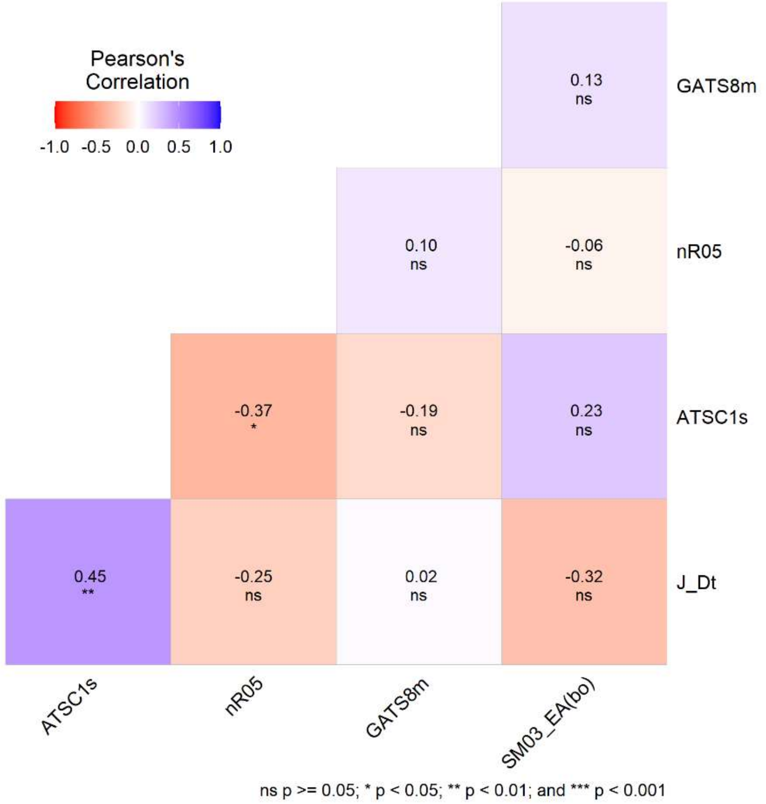

Figure S7. Correlation matrix of molecular descriptors selected in Pb^{2+} $\log\beta$ QSPR model.

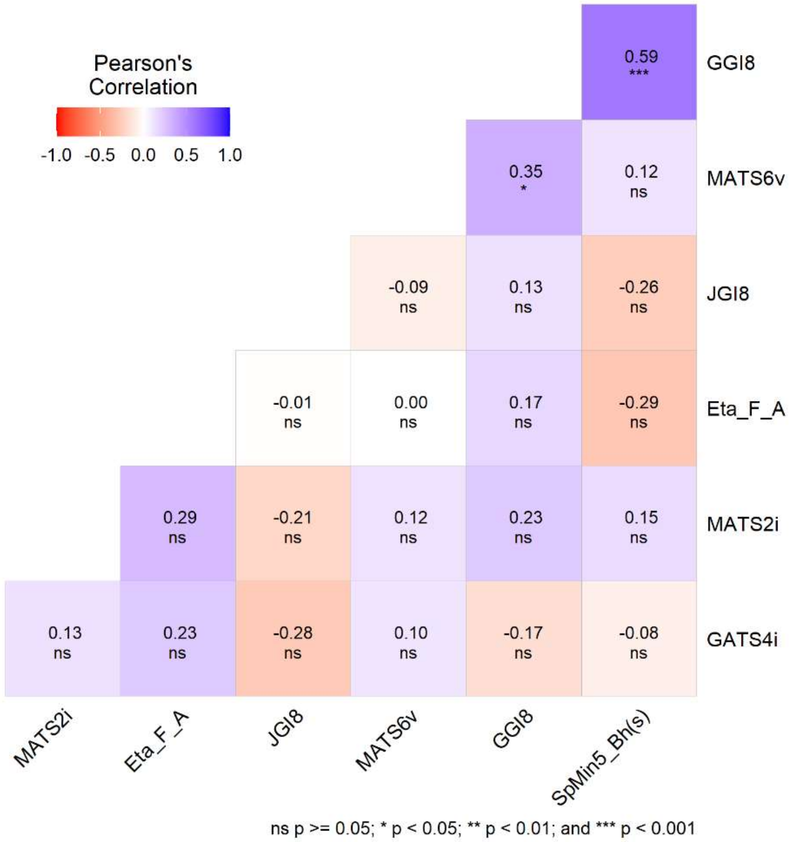

Figure S8. Correlation matrix of molecular descriptors selected in Pb^{2+} PS_{ML} QSPR model.

Table S12. Performance comparison between GA-MLR and ML models for predicting logβ of complex between Cd^{2+} and compounds.

Algorithms	R^2 *k*-fold cross-validation	Q^2 external test set
Nonlinear Regression	0.925724	0.812039
AdaBoost Regressor MLR	0.921837	0.81617
Gaussian Process Regressor	0.894938	0.805267
Random Forest Regressor	0.796154	0.80026
The best MLR model	0.9079	0.812

Table S13. Performance comparison between GA-MLR and ML models for predicting PS_{ML} of complex between Cd^{2+} and compounds.

Algorithms	R^2 *k*-fold cross-validation	Q^2 external test set
GradientBoostingRegressor	1.000	0.948434061
Random Forest Regressor	0.999722244	0.897113502
AdaBoost Regressor DT	0.999263294	0.941271334
AdaBoost Regressor MLR	0.91949539	0.879032755
Nonlinear Regression	0.91597577	0.878878122
MLP Regressor	0.915974282	0.878615791
Gaussian Process Regressor	0.906771339	0.903436134
The best MLR model	0.916	0.896166667

Table S14. Performance comparison between GA-MLR and ML models for predicting logβ of complex between Cu^{2+} and compounds.

Algorithms	R^2 *k*-fold cross-validation	Q^2 external test set
AdaBoost Regressor DT	0.984825171	0.7199684
AdaBoost Regressor MLR	0.913765445	0.82809407
Nonlinear Regression	0.908522824	0.827603944
MLP Regressor	0.908522741	0.827610512
Gaussian Process Regressor	0.908519552	0.827651378
Support Vector Regressor	0.901498761	0.808066825
The best MLR model	0.8862	0.8326

Table S15. Performance comparison between GA-MLR and ML models for predicting PS_{ML} of complex between Cu^{2+} and compounds.

Algorithms	R^2 *k*-fold cross-validation	Q^2 external test set
Gradient Boosting Regressor	0.999999904	0.709534709
AdaBoost Regressor MLR	0.89802849	0.816214429
Nonlinear Regression	0.890398658	0.879333515
MLP Regressor	0.890398605	0.879418781
Gaussian Process Regressor	0.888774511	0.87817946
Support Vector Regressor	0.884442409	0.901160709
The best MLR model	0.8669	0.859566667

Table S16. Performance comparison between GA-MLR and ML models for predicting logβ of complex between Pb^{2+} and compounds.

Algorithms	R^2 *k*-fold cross-validation	Q^2 external test set
Gradient Boosting Regressor	1.000	0.847080049
AdaBoost Regressor DT	0.95625577	0.664698943
MLP Regressor	0.90313783	0.853574424
Nonlinear Regression	0.808369698	0.630501768
The best MLR model	0.8084	0.6305

Table S17. Performance comparison between GA-MLR and ML models for predicting PS_{ML} of complex between Pb^{2+} and compounds.

Algorithms	R^2 *k*-fold cross-validation	Q^2 Test set
Nonlinear Regression	0.925724257	0.812039297
AdaBoost Regressor MLR	0.92183658	0.816170429
Gaussian Process Regressor	0.894938001	0.805266602
Random Forest Regressor	0.796154256	0.800259789
The best MLR model	0.908	0.7289

Table S18. Logβ and PS_{ML} parameter predictions for virtual chemical library based on corresponding QSPR models.

Cpd. ID	logβCd²⁺-L	logβCu²⁺-L	logβPb²⁺-L	PSCd²⁺-L	PSCu²⁺-L	PSPb²⁺-L
NEW01	7.2299	6.2157	3.3078	8.7302	27.3728	25.1919
NEW02	**9.5366**	**9.0221**	**3.1326**	**6.1456**	**13.8088**	**16.1353**
NEW03	**10.2625**	**9.6099**	**2.9345**	**7.9005**	**16.1938**	**16.9472**
NEW04	6.7047	3.4281	2.0696	13.6479	32.4903	28.5516
NEW05	6.5049	5.7129	1.7343	26.8981	47.3703	23.5674
NEW06	7.4011	6.7300	2.3939	15.6932	41.1356	24.1475
NEW07	**8.2776**	**8.8702**	**4.3037**	**4.5160**	**5.5176**	**17.9727**
NEW08	5.9427	3.9873	2.1182	14.2646	41.3550	27.8020
NEW09	5.9353	2.1858	1.3775	26.0268	39.6955	28.8286
NEW10	4.3961	6.6666	1.1218	10.6837	22.1229	25.1430
NEW11	3.8955	2.3925	2.3358	17.8985	35.3738	21.0525
NEW12	3.9942	4.7579	1.9680	21.1548	43.8542	29.9012
NEW13	6.0639	5.4509	1.5857	21.0120	26.7694	31.3223
NEW14	4.1477	4.3792	2.8353	9.7048	24.1167	25.3185
NEW15	5.9427	3.9873	2.1182	14.2646	41.3550	27.8020
NEW16	7.0546	7.4936	2.0028	18.2902	48.2008	19.2659
NEW17	8.4495	5.9013	1.2629	17.2032	43.3520	30.9070
NEW18	5.0191	2.9372	1.2920	8.1171	45.3264	23.1692
NEW19	3.6735	7.6125	2.1332	22.2732	42.8704	22.8907
NEW20	7.2263	7.9642	1.1124	22.8437	31.0370	29.7044
NEW21	**6.9122**	**5.5059**	**4.0236**	**4.7459**	**22.3069**	**16.8122**
NEW22	7.8321	8.0161	3.8116	28.0867	46.6198	27.7988
NEW23	4.7849	3.2686	2.7758	7.3873	46.4206	21.9786
NEW24	7.7830	7.4738	1.3868	12.3757	26.9581	29.6085
NEW25	3.2838	5.6766	1.1455	14.8074	26.4679	22.2587
NEW26	**7.0869**	**7.7013**	**2.5389**	**7.2298**	**34.3599**	**27.2861**
NEW27	3.4714	6.5216	2.4352	10.3017	43.2585	29.3693
NEW28	7.8051	3.6398	1.5326	13.2976	42.1579	27.8926
NEW29	2.6921	2.2182	2.0532	17.3256	17.9481	26.9962
NEW30	7.9301	4.3473	2.5967	9.3132	45.0338	19.1306
NEW31	2.4407	3.9638	1.2911	13.9341	44.1849	19.2181
NEW32	4.0845	5.3336	2.6840	23.2887	30.6075	31.1596
NEW33	2.3771	6.6561	1.7555	21.2558	31.8574	30.7486
NEW34	**6.5502**	**6.6073**	**3.4345**	**4.2357**	**28.9751**	**23.5070**
NEW35	2.0748	3.1554	2.4332	10.0122	25.0810	20.0914
NEW36	7.1972	5.4692	2.0355	16.9356	43.1862	23.0296
NEW37	6.4996	4.4118	2.0823	15.2367	38.0352	21.6537
NEW38	2.6301	7.2491	2.4218	25.4922	42.2618	19.1759
NEW39	6.2306	4.8096	1.7720	11.5485	47.3764	21.9894
NEW40	7.8763	7.3694	2.7352	20.7155	40.9639	20.3910
NEW41	4.6427	2.2374	2.7113	20.1505	35.4517	30.4258
NEW42	7.1477	4.4264	1.8435	19.8252	41.1344	25.7951
NEW43	3.6335	3.8462	2.9087	19.9432	48.8608	23.1474
NEW44	7.4535	2.9242	2.7475	13.7177	42.2348	27.4428
NEW45	3.3601	6.7990	2.9391	11.9799	39.2027	22.0011
NEW46	5.8075	5.4723	2.4170	23.0268	35.3789	24.1608
NEW47	7.0350	6.7095	2.8926	8.7249	30.4211	30.9279
NEW48	8.8210	4.2791	2.2376	21.0566	27.5683	24.8222
NEW49	1.9762	7.8223	2.2923	8.5261	28.7520	20.5893
NEW50	6.6665	2.2263	2.1803	25.4796	23.2913	27.0648
NEW51	**9.5662**	**9.2518**	**2.6083**	**8.1187**	**30.9020**	**21.5195**
NEW52	4.9879	4.1479	2.0487	24.7167	23.7333	22.4432
NEW53	6.8560	6.6958	1.0996	15.3090	31.1021	29.2352
NEW54	8.2729	3.8759	2.6596	19.1724	39.1489	27.3762

NEW55	8.1645	6.6975	1.6665	24.8623	45.0272	24.3059
NEW56	1.6543	6.4612	2.8792	6.2272	23.0518	29.5758
NEW57	1.6898	3.2218	2.1147	19.3600	16.9656	26.3104
NEW58	1.1533	2.1791	1.8404	12.4207	16.5668	20.7855
NEW59	8.7145	5.6998	1.7908	11.2607	21.9079	27.9829
NEW60	3.3158	6.6195	1.8194	7.7992	47.9358	23.8375
NEW61	4.9352	5.0315	1.6906	25.0250	39.4105	21.1388
NEW62	6.0896	5.6101	1.9721	7.9151	40.2836	19.6851
NEW63	7.5917	2.8304	2.1447	24.3250	31.4192	30.9164
NEW64	1.0187	4.2657	2.4624	17.7459	19.1249	19.5308
NEW65	2.8927	2.0359	1.2955	9.3647	29.6794	28.4441
NEW66	4.0900	7.1128	1.2212	7.2629	34.4103	23.3638
NEW67	4.5701	4.9775	1.4525	21.1858	22.9043	29.5330
NEW68	6.5826	4.6317	1.9609	15.7758	17.6500	26.2751
NEW69	4.3163	7.7149	2.4439	19.9216	18.9900	28.1609
NEW70	4.3474	7.0819	1.7218	24.6569	47.2808	25.7825
NEW71	7.2505	5.4229	1.5226	17.6207	19.1627	27.9053
NEW72	3.8564	7.2473	1.7698	25.1541	45.2664	29.2653
NEW73	8.9248	7.4060	1.6575	10.3770	34.8656	20.6891
NEW74	2.6450	7.7631	1.5381	11.2492	43.8565	26.7890
NEW75	6.6214	6.5344	1.8749	21.5455	17.4935	21.9733
NEW76	2.5577	5.5167	2.6695	10.7763	43.5086	29.1711
NEW77	4.5751	7.0862	2.3664	9.1689	20.0526	29.8286
NEW78	5.0978	5.2523	1.8707	18.8639	15.4483	26.7806
NEW79	5.0567	7.7973	2.7347	25.7360	40.9123	27.1178
NEW80	**6.9634**	**4.2819**	**3.8906**	**8.0437**	**15.6780**	**13.5199**
NEW81	5.9565	7.3462	2.7350	11.3116	27.4177	22.4807
NEW82	8.0368	4.4904	2.6003	14.1775	20.2919	28.8381
NEW83	7.4498	5.5964	1.6470	17.4808	48.1288	31.4523
NEW84	3.5829	6.2591	1.3910	23.5404	24.3348	23.8976
NEW85	4.6850	6.1152	2.9339	13.4108	43.5568	22.5463
NEW86	6.4575	4.2391	1.0849	16.1790	31.3893	29.7753
NEW87	1.4698	4.5507	2.9132	14.6397	37.1117	25.3129
NEW88	7.9420	2.5621	2.4061	8.3417	20.6945	26.4133
NEW89	2.6721	5.0474	1.5498	16.0166	24.2345	28.3751
NEW90	7.5706	6.5180	1.0139	23.9898	32.7768	29.2826
NEW91	1.6811	2.4509	1.4419	23.6841	31.2580	24.5005
NEW92	4.3572	5.2104	1.9836	19.9855	42.6401	20.5510
NEW93	3.3335	5.6488	2.9198	14.9967	24.8512	25.7150
NEW94	2.3031	7.4279	1.7588	16.2310	27.5513	22.5189
NEW95	2.4815	5.4395	1.8069	10.2280	33.5801	26.7573
NEW96	1.4007	7.8755	2.0496	18.9764	30.1319	29.1028
NEW97	6.3741	6.7618	1.4193	6.7112	28.3528	26.4166
NEW98	5.2091	4.8873	1.2829	6.8880	15.3672	21.3310
NEW99	3.1151	6.2603	2.3805	6.5489	38.4944	23.0382
NEW100	8.8038	3.0151	1.0658	23.5426	39.9104	26.8141
NEW101	1.7817	3.0886	2.0405	6.9137	28.2965	29.4521
NEW102	2.8379	5.7159	1.6366	24.8809	20.8408	31.6558
NEW103	4.1491	2.7742	2.6297	19.3234	43.7813	25.6351
NEW104	3.8282	7.1541	2.2413	18.6354	35.0084	31.7499
NEW105	2.1381	6.4219	2.5499	8.1758	25.5342	31.8863
NEW106	7.5371	4.2630	2.4058	18.1523	43.9213	25.3129
NEW107	4.2194	2.5405	1.2075	21.4832	47.0184	29.3493
NEW108	8.6902	2.9129	1.1785	23.2004	36.9826	23.8585
NEW109	7.0808	6.4475	2.9064	26.1702	42.9831	21.6406
NEW110	6.7982	7.8291	1.7667	13.2933	18.8921	26.4585
NEW111	1.9653	3.0520	2.1439	11.6289	48.0089	22.3121
NEW112	3.2786	5.2025	2.9979	22.4588	19.5708	23.3902

NEW113	5.5018	6.5269	2.5301	6.6104	17.2804	26.8373
NEW114	7.8935	5.3341	2.2297	23.0358	28.1115	31.6892
NEW115	3.8525	5.5194	2.2790	21.5286	30.7347	24.4923
NEW116	7.1269	3.3690	1.5033	14.0852	45.1590	27.0585
NEW117	2.2230	6.3289	1.9186	15.6836	24.5852	22.9292
NEW118	7.3738	2.2777	2.8597	24.5596	25.0328	30.1009
NEW119	7.8091	4.4351	2.0492	18.0294	19.5008	30.2548
NEW120	5.6282	6.5053	2.8463	7.1527	15.6546	26.1429
NEW121	3.9253	6.6915	1.6699	6.1637	32.1216	31.8265
NEW122	2.9809	6.6528	1.2116	18.3855	24.6645	20.4093
NEW123	3.1408	3.0533	2.7905	22.3401	44.0674	27.1524
NEW124	6.3213	7.6312	1.1591	11.1617	41.5585	25.1247
NEW125	4.6233	4.2032	1.2329	7.6455	39.5289	31.5880
NEW126	7.7785	6.0622	1.0006	10.1562	22.7337	21.3444
NEW127	5.7653	7.5126	2.6909	15.6622	46.4079	28.2403
NEW128	3.2376	2.7069	1.4121	18.1700	35.2912	26.6503
NEW129	7.4075	3.3918	1.4222	11.1651	23.1546	26.3452

Table S19. SMILES code of 8 new coumarin-like structures and their leverage value (h_i) computed by 6 QSPR models.

Cpd. ID	SMILES	Leverage value (h_i)					
		**AD - Logβ$_{CdL}$**	**AD - Logβ$_{CuL}$**	**AD - Logβ$_{PbL}$**	**AD - PS$_{CdL}$**	**AD - PS$_{CuL}$**	**AD - PS$_{PbL}$**
NEW02	SC1=CC=CC=C1/C=N/C2=CC3=C(C=C2)C(C)=CC(O3)=O	0.1085	0.1925	0.0916	0.2375	0.2943	0.5894
NEW03	SC1=CC=C(/C=N/C2=CC3=C(C(C)=CC(O3)=O)C=C2)C(S)=C1	0.2993	0.3159	0.0899	0.2177	0.3028	0.477
NEW07	OC1=CC=CC=C1/C=N/C2=C(O)C3=C(C(C)=CC(O3)=O)C=C2	0.1592	0.1281	0.0737	0.2529	0.4298	0.2202
NEW21	NC1=CC=CC=C1/C=N/C2=C(N)C3=C(C(C)=CC(O3)=O)C=C2	0.2133	0.1233	0.0723	0.2676	0.1899	0.2018
NEW26	CC(C(C=C1)=C2C=C1/N=C/C(C(N)=C3)=CC=C3NC)=CC(O2)=O	0.2302	0.2333	0.1024	0.2084	0.233	0.3437
NEW34	NC1=CC=CC=C1/C=N/C2=CC3=C(C(C)=CC(O3)=O)C=C2	0.1683	0.1281	0.0796	0.2546	0.2365	0.3958
NEW51	SC1=CC(SC)=CC=C1/C=N/C2=CC3=C(C(C)=CC(O3)=O)C=C2	0.1843	0.1155	0.1075	0.1393	0.5251	0.3362
NEW80	O=C(OCC1=NC=CC=C1)C2=CC3=CC=CC=C3OC2=O	0.0827	0.213	0.0772	0.4016	0.1703	0.3547
	**Critical leverage of each model (h*)**	0.6563	0.6316	0.6429	0.6486	0.6000	0.6667

Table S20. Physicochemical and absorption, distribution, metabolism, and excretion (ADME) properties of three candidates based on SwissADME tool.

**Molecule**	**NEW02**	**NEW03**	**NEW07**
Formula	$C_{17}H_{13}NO_2S$	$C_{17}H_{13}NO_2S_2$	$C_{17}H_{13}NO_4$
MW	295.36	327.42	295.29
#Heavy atoms	21	22	22
#Aromatic heavy atoms	16	16	16
Fraction Csp3	0.06	0.06	0.06
#Rotatable bonds	2	2	2
#H-bond acceptors	3	3	5

#H-bond donors	0	0	2
Molar Refractivity	88.4	95.65	85.19
TPSA	81.37	120.17	83.03
iLOGP	2.98	3.11	2.21
WLOGP	4.14	4.43	3.26
MLOGP	3.16	3.43	1.75
Consensus LogP	3.82	4.04	2.7
ESOL LogS	-4.44	-4.71	-3.6
ESOL Solubility (mg/ml)	1.07E-02	6.41E-03	7.50E-02
ESOL Class	Moderately soluble	Moderately soluble	Soluble
Ali LogS	-5.11	-6.08	-3.8
Ali Solubility (mg/ml)	2.29E-03	2.72E-04	4.73E-02
Ali Class	Moderately soluble	Poorly soluble	Soluble
Silicos-IT LogSw	-6.71	-6.8	-5.45
Silicos-IT Solubility (mg/ml)	5.76E-05	5.13E-05	1.05E-03
Silicos-IT class	Poorly soluble	Poorly soluble	Moderately soluble
Gastrointestinal absorption	Moderately	Moderately	Moderately
BBB permeant	No	No	No
Pgp substrate	No	No	No
Skin permeability logKp (cm/s)	-5.47	-5.56	-6.39
Bioavailability Score	0.55	0.55	0.55

Table S21. Molecular descriptors calculated for compounds in the training and test sets of PS_{CuL} database.

Code	Ho_Dz(p)	GGI10	P_VSA_p_2	Chi1_EA(dm)	DLS_04	LLS_01	Selection
SEN-01	59.945	0.165	65.929	15.847	0.2	0.33	Training
SEN-02	61.251	0.231	65.929	16.917	0.2	0.33	Training
SEN-03	62.556	0.298	65.929	17.988	0.2	0.33	Training
SEN-04	28.800	0.000	70.489	16.397	1.0	0.67	Training
SEN-05	29.380	0.016	70.489	16.397	1.0	0.67	Training
SEN-06	39.516	0.132	70.489	16.397	1.0	0.33	Training
SEN-07	34.794	0.099	71.102	21.397	1.0	0.50	Training
SEN-08	36.382	0.116	71.102	27.76	0.8	0.50	Training
SEN-09	38.383	0.182	71.102	23.925	0.8	0.33	Training
SEN-10	38.414	0.173	71.102	27.874	0.8	0.33	Training
SEN-11	40.640	0.173	71.102	26.257	0.6	0.33	Test
SEN-12	40.828	0.148	122.874	20.425	0.6	0.33	Training
SEN-13	29.341	0.074	100.874	10.104	1.0	0.33	Test
SEN-14	50.010	0.124	122.874	15.773	1.0	0.17	Training
SEN-15	48.869	0.182	144.874	27.002	1.0	0.33	Training
SEN-16	52.293	0.190	115.101	24.699	1.0	0.33	Training
SEN-17	42.684	0.223	86.662	24.932	0.6	0.17	Training
SEN-18	46.544	0.215	86.662	16.397	1.0	0.17	Training
SEN-19	44.289	0.289	86.662	26.284	0.5	0.17	Training
SEN-20	47.461	0.313	86.662	26.257	0.6	0.17	Training
SEN-21	58.761	0.396	86.662	27.874	0.8	0.17	Training
SEN-22	60.355	0.495	86.662	29.225	0.8	0.17	Test

SEN-23	28.181	0.029	60.002	12.466	0.6	0.50	Excluded
SEN-24	18.548	0.000	99.561	1.651	0.4	0.67	Training
SEN-25	15.903	0.000	62.793	2.344	0.4	1.00	Prediction
SEN-26	50.286	0.081	227.407	24.742	0.3	0.17	Training
SEN-27	55.724	0.164	238.407	27.549	0.4	0.17	Test
SEN-28	49.099	0.081	121.855	22.035	0.6	0.17	Training
SEN-29	54.541	0.164	132.855	24.843	0.7	0.17	Training
SEN-30	45.424	0.081	187.650	21.553	0.5	0.00	Training
SEN-31	50.876	0.131	198.650	24.361	0.5	0.00	Training
SEN-32	47.592	0.297	86.6620	16.397	1.0	0.17	Test
SEN-33	48.509	0.354	86.662	26.257	0.5	0.17	Training
SEN-34	37.427	0.115	166.301	5.835	0.4	0.33	Training
SEN-35	40.081	0.206	219.322	10.434	0.4	0.17	Training
SEN-36	33.719	0.050	15.325	0.000	0.2	0.50	Training
SEN-37	18.966	0.000	15.325	0.000	0.5	0.83	Test
SEN-38	27.619	0.000	33.606	0.000	0.5	0.50	Test
SEN-39	43.175	0.066	66.605	9.103	0.7	0.17	Training
SEN-40	59.515	0.099	99.605	17.525	0.9	0.33	Training
SEN-41	49.467	0.273	76.842	11.628	0.4	0.17	Training
SEN-42	53.874	0.380	89.881	16.982	0.9	0.17	Test
SEN-43	70.036	0.380	91.49	18.018	0.8	0.17	Training
SEN-44	72.718	0.363	91.49	18.018	0.8	0.17	Training
SEN-45	43.545	0.248	73.21	13.954	0.5	0.17	Training

Table S22. Molecular descriptors calculated for compounds in the training and test sets of PS_{CdL} database.

Code	J_B(s)	GATS8i	JGI8	SpMax4_Bh(m)	SpMin3_Bh(s)	SpMin5_Bh(s)	DLS_01	Selection
SEN-01	420.2345	1.004	0.006	3.737	1.524	1.353	0.5	Training
SEN-02	439.4922	1.002	0.008	3.737	1.524	1.356	0.5	Training
SEN-03	459.1729	1.001	0.009	3.737	1.524	1.358	0.5	Training
SEN-04	44.5262	1.029	0.008	3.150	1.079	0.923	1.0	Training
SEN-05	33.2807	0.970	0.007	3.334	1.122	0.853	1.0	Test
SEN-06	89.4155	0.743	0.016	3.568	1.296	1.121	1.0	Training
SEN-07	45.7187	1.006	0.011	3.526	1.136	0.928	1.0	Training
SEN-08	41.6672	1.061	0.009	3.534	1.105	1.034	1.0	Training
SEN-09	54.5813	1.045	0.012	3.586	1.128	1.022	1.0	Training
SEN-10	54.5631	1.080	0.011	3.613	1.140	1.029	1.0	Training
SEN-11	52.9249	0.995	0.011	3.599	1.101	1.011	1.0	Training
SEN-12	54.8432	0.953	0.006	3.652	1.148	1.048	1.0	Training
SEN-13	55.9514	0.941	0.008	3.557	1.109	0.967	1.0	Training
SEN-14	140.9681	1.009	0.006	3.676	1.287	1.172	1.0	Training
SEN-15	68.7549	0.838	0.009	3.654	1.152	1.048	1.0	Training
SEN-16	77.8878	0.914	0.008	3.774	1.168	1.059	1.0	Training
SEN-17	57.8869	1.051	0.008	3.852	1.181	1.034	1.0	Training
SEN-18	94.9091	1.027	0.009	3.709	1.323	1.198	1.0	Training
SEN-19	63.9171	1.157	0.008	4.016	1.192	1.034	0.75	Test
SEN-20	62.715	0.992	0.009	3.864	1.179	1.027	1.0	Training

SEN-21	108.3086	1.175	0.009	3.882	1.318	1.210	0.5	Training
SEN-22	116.4965	1.234	0.009	4.059	1.319	1.221	0.5	Training
SEN-23	33.0552	0.934	0.007	3.500	1.041	0.414	1.0	Training
SEN-24	17.1538	0.757	0.020	2.889	0.360	0.000	1.0	Training
SEN-25	15.2931	0.849	0.020	2.879	0.341	0.000	1.0	Training
SEN-26	55.6744	0.944	0.009	3.852	1.033	0.483	0.75	Training
SEN-27	66.0329	1.116	0.008	3.854	1.116	0.497	0.5	Training
SEN-28	63.9375	0.979	0.008	3.827	1.053	0.942	0.5	Training
SEN-29	74.9841	1.104	0.007	3.836	1.054	1.007	0.25	Test
SEN-30	50.1821	0.963	0.007	3.780	0.942	0.529	0.75	Training
SEN-31	60.04	1.124	0.006	3.787	1.009	0.530	0.75	Test
SEN-32	82.2979	1.012	0.010	3.704	1.431	1.323	1.0	Training
SEN-33	59.9802	0.924	0.010	3.847	1.247	1.032	0.5	Training
SEN-34	45.8078	1.029	0.005	3.594	1.072	0.449	1.0	Test
SEN-35	51.5971	0.948	0.005	3.598	1.114	0.452	1.0	Training
SEN-36	170.7217	1.019	0.005	3.737	1.524	1.258	0.75	Training
SEN-37	25.6378	0.879	0.000	3.617	1.144	0.245	0.75	Training
SEN-38	43.2814	1.027	0.004	3.884	1.144	0.972	0.75	Training
SEN-39	69.3908	0.908	0.005	3.884	1.147	1.144	0.75	Test
SEN-40	106.4015	0.855	0.004	3.884	1.191	1.144	0.75	Test
SEN-41	80.8224	1.056	0.010	3.757	1.338	1.010	0.5	Training
SEN-42	106.0039	1.064	0.007	3.729	1.281	1.018	0.5	Training
SEN-43	172.7874	1.027	0.006	3.884	1.520	1.452	0.5	Test
SEN-44	185.4518	1.034	0.005	3.884	1.520	1.498	0.5	Training
SEN-45	69.447	1.066	0.009	3.884	1.377	1.144	0.5	Training

Table S23. Molecular descriptors calculated for compounds in the training and test sets of PS_{PbL} database.

Code	MATS6v	MATS2i	GATS4i	GGI8	JGI8	SpMin5_Bh(s)	Eta_F_A	Selection
SEN-01	0.001	-0.167	1.087	0.346	0.006	1.353	0.365	Training
SEN-02	0.011	-0.169	1.074	0.444	0.008	1.356	0.367	Training
SEN-03	0.032	-0.171	1.048	0.543	0.009	1.358	0.37	Training
SEN-04	-0.012	-0.077	1.365	0.099	0.008	0.923	0.802	Training
SEN-05	-0.155	-0.108	0.976	0.107	0.007	0.853	0.728	Test
SEN-06	-0.012	-0.002	0.557	0.46	0.016	1.121	0.713	Training
SEN-07	-0.033	-0.066	1.05	0.254	0.011	0.928	0.71	Training
SEN-08	0.033	-0.117	1.096	0.288	0.009	1.034	1.051	Training
SEN-09	-0.06	-0.053	1.154	0.369	0.012	1.022	1.101	Training
SEN-10	-0.01	-0.053	1.194	0.361	0.011	1.029	1.095	Training
SEN-11	-0.027	-0.166	0.995	0.361	0.011	1.011	1.157	Training
SEN-12	-0.051	-0.161	1.157	0.198	0.006	1.048	1.044	Training
SEN-13	0.094	-0.173	1.177	0.14	0.008	0.967	0.628	Training
SEN-14	0.016	-0.192	1.071	0.189	0.006	1.172	0.624	Training
SEN-15	-0.11	-0.036	1.243	0.346	0.009	1.048	1.1	Training
SEN-16	-0.017	0.141	1.057	0.427	0.008	1.059	1.128	Training
SEN-17	-0.051	-0.176	1.179	0.304	0.008	1.034	1.208	Training
SEN-18	-0.115	-0.145	1.062	0.378	0.009	1.198	0.848	Training

SEN-19	0.039	-0.137	1.176	0.395	0.008	1.034	1.097	Test
SEN-20	-0.026	-0.18	0.999	0.385	0.009	1.027	1.248	Training
SEN-21	0.028	-0.145	1.111	0.55	0.009	1.21	1.057	Training
SEN-22	0.093	-0.12	1.104	0.624	0.009	1.221	0.958	Training
SEN-23	-0.072	-0.196	1.148	0.127	0.007	0.414	1.039	Training
SEN-24	-0.135	-0.305	1.05	0.041	0.02	0	0.929	Training
SEN-25	-0.127	-0.313	1.034	0.02	0.02	0	0.908	Training
SEN-26	0.103	0.086	1.119	0.345	0.009	0.483	1.149	Training
SEN-27	0.104	0.073	1.16	0.278	0.008	0.497	1.119	Training
SEN-28	0.028	-0.225	0.982	0.369	0.008	0.942	1.046	Training
SEN-29	0.037	-0.208	1.022	0.303	0.007	1.007	1.023	Test
SEN-30	0.048	0.107	1.127	0.254	0.007	0.529	1.127	Training
SEN-31	0.056	0.091	1.165	0.188	0.006	0.53	1.097	Test
SEN-32	-0.093	-0.083	1.077	0.509	0.01	1.323	0.895	Training
SEN-33	-0.004	-0.134	1.001	0.558	0.01	1.032	1.281	Training
SEN-34	0.038	-0.282	1.099	0.132	0.005	0.449	1.128	Test
SEN-35	0.092	-0.188	1.206	0.181	0.005	0.452	1.178	Training
SEN-36	0	-0.21	1.019	0.148	0.005	1.258	0.102	Training
SEN-37	-0.216	-0.12	0.979	0	0	0.245	1.025	Training
SEN-38	-0.025	-0.128	1.073	0.129	0.004	0.972	1.196	Training
SEN-39	-0.146	-0.003	1.218	0.156	0.005	1.144	1.097	Test
SEN-40	-0.103	0.037	1.264	0.156	0.004	1.144	1.037	Test
SEN-41	0.03	-0.086	1.167	0.895	0.01	1.01	1.407	Training
SEN-42	-0.05	0.027	1.04	0.437	0.007	1.018	1.165	Training
SEN-43	-0.028	-0.046	1.011	0.511	0.006	1.452	1.02	Test
SEN-44	-0.016	-0.055	1.01	0.462	0.005	1.498	0.984	Training
SEN-45	-0.013	0.046	1.157	0.401	0.009	1.144	1.212	Training

Table S24. Molecular descriptors calculated for compounds in the training and test sets of Logβ_{CuL} database.

Code	nHM	ATSC4e	MATS1v	MATS6p	MATS7p	GATS5p	P_VSA_ MR_7	SM07_ AEA(ed)	O-057	NssCH2	B06[C-O]	Selection
L1	0.500	0.143	0.485	0.292	0.140	0.651	0.000	0.547	0.000	0.000	1.000	Training
L2	0.500	0.112	0.338	0.060	0.082	0.496	0.199	0.519	0.000	0.000	0.000	Training
L3	0.500	0.119	0.447	0.413	0.247	0.506	0.000	0.666	0.000	0.000	1.000	Training
L4	0.500	0.126	0.620	0.498	0.428	0.689	0.000	0.694	0.000	0.000	0.000	Training
L5	0.500	0.065	0.928	0.111	0.264	0.512	0.241	0.553	0.000	0.063	0.000	Training
L6	0.500	0.078	0.932	0.600	0.407	0.800	0.070	0.711	0.250	0.000	1.000	Test
L7	0.500	0.047	0.616	0.592	0.329	0.759	0.195	0.481	0.000	0.000	0.000	Test
L8	0.500	0.047	0.359	0.745	0.035	0.728	0.070	0.604	0.000	0.000	0.000	Training
L10	0.500	0.101	0.895	0.766	0.193	0.645	0.070	0.549	0.250	0.000	1.000	Training
L11	0.500	0.078	0.895	0.663	0.208	0.680	0.070	0.553	0.250	0.000	0.000	Training
L12	0.500	0.029	0.709	0.734	0.156	0.710	0.070	0.481	0.000	0.000	0.000	Training
L13	0.500	0.045	0.540	0.767	0.348	0.895	0.195	0.521	0.000	0.000	0.000	Training
L14	0.500	0.065	0.540	0.144	0.531	0.707	0.120	0.547	0.000	0.000	0.000	Test
L15	0.500	0.059	0.540	0.136	0.328	0.556	0.125	0.547	0.000	0.000	0.000	Training
L16	0.500	0.097	0.886	0.386	0.388	0.812	0.195	0.651	0.250	0.000	0.000	Training

L17	0.500	0.089	0.570	0.441	0.335	1.000	0.195	0.656	0.250	0.063	0.000	Training
L18	0.500	0.091	0.675	0.361	0.348	0.944	0.195	0.673	0.250	0.000	0.000	Training
L19	0.500	0.202	0.481	0.380	0.291	0.671	0.070	0.729	0.250	0.000	1.000	Training
L21	0.500	0.092	0.793	0.379	0.400	0.630	0.000	0.679	0.250	0.000	1.000	Training
L22	0.500	0.073	0.262	0.189	0.286	0.605	0.120	0.554	0.000	0.000	0.000	Training
L23	0.500	0.269	0.658	0.601	0.196	0.783	0.070	0.771	0.500	0.250	1.000	Training
L24	0.500	0.144	0.658	0.549	0.345	0.669	0.070	0.715	0.250	0.000	1.000	Training
L26	0.500	0.112	0.203	0.390	0.219	0.464	0.245	0.634	0.000	0.000	0.000	Test
L27	0.500	0.105	0.570	0.000	0.285	0.577	0.199	0.511	0.000	0.000	0.000	Training
L28	0.000	0.192	0.637	0.418	0.383	0.694	0.000	0.602	0.000	0.625	0.000	Training
L29	0.000	0.212	0.624	0.323	0.302	0.697	0.000	0.602	0.000	0.688	1.000	Test
L30	0.000	0.211	0.574	0.431	0.314	0.700	0.000	0.602	0.000	0.750	0.000	Training
L31	0.500	0.653	0.312	0.614	0.218	0.767	0.000	0.713	1.000	0.563	0.000	Training
L32	0.500	0.553	0.443	0.550	0.223	0.770	0.022	0.763	1.000	0.563	0.000	Training
L33	0.500	0.172	0.253	0.767	0.285	0.110	0.012	0.836	0.000	0.000	0.000	Training
L34	0.500	0.223	0.667	0.802	0.285	0.118	0.000	0.836	0.000	0.063	0.000	Training
L35	0.500	0.251	0.266	0.078	0.376	0.799	0.000	0.558	0.000	0.188	1.000	Training
L36	0.500	0.390	0.430	0.169	0.464	0.716	0.375	0.748	0.000	0.250	1.000	Training
L37	0.000	0.060	0.371	0.376	0.236	0.835	0.736	0.721	0.000	0.000	0.000	Training
L38	0.000	0.141	0.599	0.328	0.241	0.690	1.000	0.762	0.000	0.000	0.000	Training
L39	0.000	0.195	0.595	0.432	0.289	0.705	0.741	0.973	0.000	0.125	0.000	Training
L40	0.000	0.036	0.451	0.220	0.187	0.671	0.125	0.585	0.000	0.000	0.000	Training
L41	0.000	0.287	0.350	0.574	0.277	0.868	0.198	0.697	0.000	0.188	1.000	Test
L42	0.000	0.256	0.637	0.295	0.330	0.878	0.120	0.575	0.250	0.125	1.000	Training
L43	0.000	0.326	0.582	0.351	0.253	0.889	0.120	0.595	0.250	0.188	1.000	Training
L44	0.000	0.252	0.637	0.310	0.406	0.859	0.120	0.567	0.250	0.125	1.000	Training
L45	1.000	0.091	0.295	0.058	0.398	0.668	0.230	0.581	0.000	0.500	0.000	Training
L46	0.500	0.136	0.312	0.328	0.247	0.747	0.125	0.510	0.000	0.688	0.000	Test
L47	1.000	0.148	0.338	0.277	0.240	0.719	0.250	0.581	0.000	0.750	0.000	Training
L48	0.000	0.459	0.675	0.891	0.285	0.009	0.000	0.713	0.000	0.188	0.000	Training
L50	0.000	0.630	0.662	0.163	0.276	0.838	0.258	0.842	0.750	0.125	1.000	Training
L51	0.000	1.000	0.430	0.351	0.286	0.790	0.220	1.000	0.000	1.000	1.000	Training
L52	0.000	0.768	0.511	0.391	0.280	0.771	0.220	1.000	0.000	0.750	1.000	Test
L53	0.000	0.071	0.519	0.389	0.285	0.000	0.000	0.458	0.250	0.063	0.000	Training
L54	0.000	0.182	0.422	0.452	0.525	0.719	0.000	0.489	0.500	0.125	0.000	Training
L55	0.000	0.335	0.329	0.493	0.551	0.796	0.000	0.556	0.750	0.188	0.000	Training
L56	0.000	0.090	0.270	0.393	0.477	0.806	0.000	0.467	0.250	0.188	0.000	Test
L57	0.000	0.460	0.207	0.507	0.234	0.700	0.000	0.596	1.000	0.375	0.000	Training
L58	0.000	0.017	0.063	0.389	0.285	0.000	0.000	0.000	0.000	0.125	0.000	Training
L59	0.000	0.075	0.152	0.471	0.266	0.802	0.000	0.058	0.000	0.500	0.000	Training
L60	0.000	0.286	0.954	0.900	0.678	0.305	0.000	0.606	0.500	0.063	0.000	Training
L61	0.000	0.116	0.835	0.222	0.315	0.840	0.000	0.618	0.250	0.063	1.000	Training
L62	0.000	0.142	0.962	0.260	0.236	0.894	0.000	0.688	0.250	0.063	1.000	Test
L63	0.000	0.288	0.785	0.497	0.572	0.600	0.000	0.604	0.500	0.125	0.000	Training
L64	0.000	0.245	1.000	0.865	0.688	0.460	0.000	0.606	0.250	0.063	0.000	Test
L65	0.000	0.132	0.384	0.741	0.631	0.866	0.000	0.606	0.250	0.063	0.000	Training

L66	0.000	0.072	0.700	1.000	1.000	0.563	0.000	0.521	0.250	0.000	0.000	Test
L67	0.000	0.086	0.367	0.271	0.212	0.728	0.000	0.664	0.250	0.438	0.000	Training
L68	0.000	0.085	0.249	0.319	0.000	0.783	0.000	0.745	0.000	0.000	1.000	Test
L69	0.000	0.131	0.295	0.402	0.185	0.699	0.000	0.750	0.000	0.375	1.000	Training
L70	0.000	0.000	0.000	0.389	0.285	0.000	0.125	0.270	0.000	0.000	0.000	Training
L71	0.000	0.193	0.371	0.315	0.292	0.683	0.000	0.489	0.000	0.375	1.000	Training
L72	0.000	0.139	0.574	0.544	0.211	0.678	0.134	0.724	0.250	0.000	1.000	Training
L74	0.000	0.181	0.481	0.461	0.350	0.712	0.134	0.690	0.250	0.000	1.000	Test
L75	0.000	0.165	0.477	0.431	0.283	0.771	0.134	0.668	0.250	0.063	1.000	Training
L76	0.000	0.205	0.481	0.557	0.215	0.712	0.134	0.736	0.250	0.000	1.000	Training

Table S25. Molecular descriptors calculated for compounds in the training and test sets of Logβ_{CdL} database.

Code	RBN	SPI	H_D/Dt	MATS1m	MATS2m	MATS6i	Selection
L77	0	0	274.8	0.084	-0.247	0.042	Training
L78	0	0	274.8	0.096	-0.272	0.004	Training
L79	0	0	274.8	0.093	-0.301	-0.155	Training
L80	3	4.973	386.04	0.092	-0.224	-0.03	Training
L81	3	4.973	386.04	0.095	-0.243	-0.126	Test
L82	6	8.344	513.279	0.068	-0.183	-0.015	Training
L83	0	0	1518.073	0.058	-0.061	0.112	Training
L84	2	6.869	1726.326	0.055	-0.045	0.175	Training
L85	0	0	497.595	0.085	-0.26	-0.095	Training
L86	3	5.633	644.129	0.086	-0.201	-0.089	Test
L87	0	0	274.8	0.075	-0.243	0.024	Training
L88	6	0	794.85	0.084	-0.077	0.005	Training
L89	6	0	827.052	0.084	-0.077	0.18	Training
L90	1	0	167.067	0.053	-0.152	-0.026	Training
L91	2	10.096	296.789	-0.019	0	0.17	Test
L92	4	20.193	556.289	-0.028	0.302	0.194	Training
L93	2	8.426	271.178	-0.023	0.045	0.309	Test
L94	2	8.426	271.178	-0.012	-0.001	0.175	Training
L95	4	18.621	515.631	-0.014	0.287	0.177	Training
L53	1	2.804	10	-0.183	0.055	0	Training
L96	2	5.586	28	-0.111	0.14	0.273	Test
L65	3	6.386	36	-0.099	0.088	0.216	Test
L97	3	6.28	36	-0.099	0.088	0.237	Training
L98	3	5.197	28	-0.111	0.036	0.201	Training
L99	4	5.865	36	-0.099	-0.001	-0.036	Training
L61	3	6.317	115.133	-0.059	-0.053	-0.145	Training
L62	3	7.542	268.96	-0.037	-0.009	-0.056	Test
L100	6	8.542	66	-0.112	0.005	-0.025	Training
L60	3	6.386	36	-0.155	0.096	0.242	Training
L101	4	7.139	45	-0.125	0.07	0.168	Training
L102	2	4.504	21	-0.172	-0.042	0.176	Training
L103	2	5.586	28	-0.144	0.09	0.22	Training
L104	3	7.901	132.467	-0.087	-0.002	-0.185	Training

L60	3	6.386	36	-0.155	0.096	0.242	Training
L42	3	5.85	85.533	-0.065	-0.018	0.027	Training
L105	4	5.865	36	0.015	-0.242	-0.173	Training
L106	2	4.504	21	-0.216	-0.16	0.284	Training
L107	3	6.386	36	-0.243	-0.04	0.485	Training
L108	4	5.865	36	-0.125	-0.047	0.163	Test
L109	2	2.79	53.567	-0.019	-0.111	0.156	Training

Table S26. Molecular descriptors calculated for compounds in the training and test sets of $Log\beta_{PbL}$ database.

**Code**	**nR05**	**J_Dt**	**ATSC1s**	**GATS8m**	**SM03_EA(bo)**	**Selection**
L110	0	0.8391	11.339	1.59	4.477	Training
L111	2	0.589	6.94	1.195	4.698	Training
L112	0	0.4013	3.972	0.823	4.321	Training
L113	0	4.156	40.64	1.304	3.892	Training
L114	0	1.0879	20.917	0.27	4.111	Test
L115	2	1.0881	4.336	1.163	3.951	Test
L116	1	0.9681	6.736	0.942	4.677	Training
L117	0	1.0123	18.826	0.479	3.761	Training
L118	1	1.039	6.531	0.972	3.98	Test
L119	1	1.0501	6.805	1.05	4.301	Training
L120	0	1.3229	14.651	0	3.761	Training
L121	2	1.0323	5.054	0.532	3.332	Training
L122	0	1.2488	6.74	0	3.892	Training
L123	0	0.834	16.453	1.285	5.278	Training
L124	0	1.0575	11.191	1.087	4.27	Test
L125	2	1.1976	8.328	1.358	3.951	Training
L126	0	1.1272	10.289	0.681	4.551	Training
L127	0	0.4182	24.136	1.036	6.048	Training
L128	2	0.6673	2.799	1.212	4.227	Training
L129	2	0.3151	2.778	1.317	5.056	Test
L130	1	1.0532	4.055	1.035	3.951	Training
L131	2	1.2558	20.217	1.11	4.635	Training
L132	0	1.1163	7.505	0.693	4.159	Training
L133	0	1.0585	6.621	1.305	4.62	Training
L134	0	0.5882	7.364	1.152	5.231	Training
L135	0	1.5163	13.546	1.522	4.477	Training
L136	0	0.9409	12.036	0.977	2.565	Training
L137	0	1.2752	68.513	0.392	5.112	Training
L138	0	0.8093	21.01	0.96	5.308	Training
L139	2	0.6997	5.296	1.168	4.407	Training
L140	2	0.5563	6.386	1.018	4.718	Test
L6	0	0.8135	11.613	1.98	4.469	Training
L141	1	0.853	8.56	2.788	4.259	Training
L142	0	1.4722	12.277	1.689	4.099	Test
L143	0	1.5138	11.583	2.266	4.073	Training
